# Novel Host Protein TBC1D16, a GTPase Activating Protein of Rab5C, Inhibits Prototype Foamy Virus Replication

**DOI:** 10.3389/fimmu.2021.658660

**Published:** 2021-07-22

**Authors:** Jun Yan, Yingcheng Zheng, Peipei Yuan, Shanshan Wang, Song Han, Jun Yin, Biwen Peng, Zhi Li, Yan Sun, Xiaohua He, Wanhong Liu

**Affiliations:** ^1^ Hubei Province Key Laboratory of Allergy and Immunology, Department of Immunology, School of Basic Medical Sciences, Wuhan University, Wuhan, China; ^2^ Department of Immunology, School of Basic Medical Sciences, Hubei University of Medicine, Shiyan, China; ^3^ Department of Physiology, School of Basic Medical Sciences, Wuhan University, Wuhan, China; ^4^ College of Life Sciences, Shanxi Normal University, Xi’an, China; ^5^ Department of Pathophysiology, School of Basic Medical Sciences, Wuhan University, Wuhan, China; ^6^ Shenzhen Research Institute, Wuhan University, Shenzhen, China

**Keywords:** prototype foamy virus (PFV), TBC1D16, Rab5C, viral replication, type I IFN

## Abstract

Prototype foamy virus (PFV) is a member of the oldest family of retroviruses and maintains lifelong latent infection in the host. The lifelong latent infection of PFV may be maintained by the restriction factors of viral replication in the host. However, the mechanisms involved in PFV latent infection are poorly understood. Here, we found that TBC1D16, a TBC domain-containing protein, is significantly down-regulated after PFV infection. Tre2/Bub2/Cdc16 (TBC) domain-containing proteins function as Rab GTPase-activating proteins (GAPs) and are participates in the progression of some diseases and many signaling pathways. However, whether TBC proteins are involved in PFV replication has not been determined. Here, we found that TBC1D16 is a novel antiviral protein that targets Rab5C to suppress PFV replication. Overexpression TBC1D16 inhibited the transcription and expression of Tas and Gag, and silencing TBC1D16 enhanced the PFV replication. Moreover, the highly conserved amino acid residues R494 and Q531 in the TBC domain of TBC1D16 were essential for inhibiting PFV replication. We also found that TBC1D16 promoted the production of PFV-induced IFN-β and the transcription of downstream genes. These results suggest that TBC1D16 might be the first identified TBC proteins that inhibited PFV replication and the mechanism by which TBC1D16 inhibited PFV replication could provide new insights for PFV latency.

## Introduction

Foamy viruses (FVs; also known as spumaviruses) are christened because they induce the endoplasmic reticulum forming voids and produce foam-like lesions when these viruses replicate in adherent cell cultures of epithelial or fibroblastoid origin (1). The first human foamy virus (HFV) isolate was obtained from cultures of a human nasopharyngeal carcinoma and was renamed prototype foamy virus (PFV) based upon sequence identity with chimpanzee FVs (2). Moreover, PFV appears to be nonpathogenic and maintains a lifelong infection in the host (3). PFV has a common genome structure of retroviruses, two long terminal repeats (LTRs) at the 5’ and 3’ ends, which directs the expression of structural genes and some regulatory genes (4). Unlike other retroviruses, PFV has an internal promoter (IP) in the coding region of the Env protein that directs the expression of the accessory proteins genes *Tas* and *Bet* (5). The transactivator of PFV, Tas (also known as Bel1), is a 300-amino-acid phosphoprotein that contains an acidic transcription activation domain at its carboxyl terminus and a centrally located DNA binding domain (6). As a DNA binding protein, Tas binds to the LTR and IP promoters of PFV activating and regulating the transcription and replication of the virus (7). The structural genes of PFV are *pol*, *gag*, and *env*, which respectively encode viral capsid protein (Gag), reverse transcriptase (Pol) and viral glycoprotein envelope (Env) (8). Gag protein plays distinct roles during the early and late stages of viral replication (9). For example, Gag protein is involved in the synthesis of viral capsid protein (10). Gag protein interacts with Env protein to maintain viral capsid morphology and reverse transcription efficiency to assemble viral capsids (11). Gag has high affinity with DNA and RNA, with a nuclear localization signal, and participates in the process of viral gene reverse transcription and host gene integration (12). These characteristics are quite different from those of other retroviruses and could be key factors in PFV latent infection and nonpathogenicity.

According to previous reports, some cellular factors and physiological processes can regulate the transcription and replication of PFV at almost any step and counteract or facilitate PFV infection by acing on distinct stages of the viral life cycle. Human innate antiretroviral defense factor-APOBEC3 functions as a potent inhibitor of PFV by interacting with the Bet of PFV (13). N-Myc interactor (Nmi) inhibits PFV replication by interacting with Tas and sequestering it in the cytoplasm (14). PHF11 inhibits the expression of Tas and thus inhibits the subsequent activation of the PFV LTR promoter (15). Since Tetherin is an interferon-induced protein, it inhibits the release and infectivity of PFV, and its transmembrane domain and GPI anchor are vital for antiviral activity (16). Previously, it was reported that Pirh2 interacted with Tas and suppressed its expression *via* the ubiquitin-proteasome pathway to inhibit PFV replication (17). It was also found that PFV infection could induce autophagy to affect the replication of PFV (18). However, to date, the mechanism of latent infection with PFV is still unclear. Therefore, it is critical to further identify new host proteins to understand PFV replication strategies.

The Tre-2/Bub2/Cdc16 (TBC) domain was first identified and designated as Tbc1, which is homologous to regions of the *tre-2* oncogene and yeast regulators of mitosis, BUB2 and cdc16 (19). The TBC domain of TBC domain-containing proteins (TBC proteins) is now widely recognized as a conserved protein domain composing of approximately 200 amino acids (20). Some TBC proteins have been implicated in multiple biological processes, such as cancer (21), disease (22), cell signaling (23) and other cellular events (24). Recent studies have shown that some TBC proteins regulate the life cycles of various viruses. TBC1D20 overexpression interferes with the early transport of human immunodeficiency virus-1 (HIV-1) envelope proteins, hindering the processing of the envelope and reducing its binding to detergent-resistant membranes, thereby leading to a reduction in the infectivity of HIV-1 virion-like particles (VLPs) (25). There is an interaction between TBC1D20 and the N terminus of hepatitis C virus (HCV) nonstructural protein NS5A (26), which is necessary for efficient HCV replication (27). As a Rab GTPase-activating protein (GAP) of Rab1, TBC1D20 is recruited to LDs and affects lipid droplet metabolism to promote the viral life cycle by interacting with NS5A (28). Hence, this finding raises interest regarding whether other TBC proteins have antiviral activity and whether TBC proteins are involved in the regulation of PFV infection.

In this study, we identified a novel antiviral TBC protein, TBC1D16, which acts as a host factor to inhibit the replication of PFV. We found that TBC1D16 is a GAP of Rab5C and inhibits PFV replication by interacting with Rab5C. In particular, R494 and Q531 in the TBC domain of TBC1D16 are the key locus for inhibiting PFV replication. Moreover, we demonstrated that TBC1D16 promotes PFV-induced IFN-β production and the transcription of downstream genes. This study revealed that TBC1D16 may be a novel negative factor act as inhibiting PFV replication, providing new insights into the understanding of PFV latent infection.

## Materials and Methods

### Cells

HEK293T cells, HT1080 cells, BHK-21 cells and THP-1 cells were stored by our laboratory. HEK293T cells were maintained in Dulbecco’s modified Eagle medium (HyClone; Cat No. SH30022.01). HT1080 cells and BHK-21 cells were maintained in minimum Eagle medium (HyClone; Cat No. SH30024.01). THP-1 cells were maintained in RPMI-1640 medium (HyClone; Cat No. SH30809.01). These cells were all supplemented with 10% (vol/vol) fetal bovine serum (FBS, Biological Industries, Lot No. 1719426) and penicillin (100 U/ml)/streptomycin (100 mg/ml) (BioSharp, Cat No. BL505A) at 37°C in a humidified atmosphere containing 5% CO_2_.

### PFV Preparation

PFV was prepared by transfecting HEK293T cells with the provirus plasmid pHSRV13 using the PEI transfection reagent (29). After 48h transfection, cells with culture medium were freeze-thawed for three cycles to release viruses. To prepare cell-free virus stocks, the culture supernatant was centrifuged at 12,000 rpm for 20 min at 4°C and filtered through a 0.22 μm-pore-size filter membrane. HT1080 cells were infected with the obtained PFV virus solution for at least 48 h, and repeat the above operation to obtain a higher concentration of PFV virus solution. In order to further increase the concentration of virus stock, the PFV virions obtained from HT1080 cells were re-infected with new HT1080 cells for at least 48 h. And then the cells with culture medium were also freeze-thawed for three cycles, centrifuged (12,000 rpm for 20 min at 4°C) and filtered through filter membrane (0.22 μm-pore-size) to obtain a high-concentration cell-free PFV virus solution and stored at −80°C. The PFV obtained by two infections of HT1080 cells was used in subsequent experiments. We seeded HT1080 cells into 96-well plates and infected them with viruses for 2 h incubation. Then, we replaced the supernatant with growth medium (with 10% FBS), and the cells were maintained for 48 h.

On the other hand, we prepared the Mock stimulus by using the same method as the PFV preparation. We added the same amount of PEI transfection reagent as the PFV group to HEK293T cells, but without the PFV precursor DNA pHSRV13. Then, following the method of preparing PFV, we obtained the cell freeze-thaw supernatant without PFV virus particles as a stimulus for the Mock group.

The virus titers were calculated as 50% tissue culture infectious doses (TCID50) (30). The HT1080 cells were seeded in a 96-well cell culture plate at 2×10^4^ cells per well, and cultured in a CO_2_ incubator at 37°C for 24 h. The PFV virus stocks were serially diluted 10 gradients in a 10-fold gradient with serum-free MEM medium. After culturing the cells for 24 h, discard the medium and add 50 μl of diluted virus to each well, and set up 8 replicate wells for each concentration. At the same time, a group of uninfected cells was set as a control. The cells were incubated at 37°C for 2 h, then the virus supernatant was discarded and 100 μl cell maintenance medium was added to each well. The 96-well plate was cultured continuously for 7 days, and the number of cytopathic wells was observed and recorded every day. Calculate the TCID50 of the virus liquid according to the Karber method.

### Viral Infection

HEK293T or HT1080 cells were seeded into 12-well plates and cultured until 80% confluency was reached. Then, the cells were infected with PFV (third generation) at a MOI of 0.5 for 2 h. The cells were washed once with phosphate-buffered saline (PBS) (HyClone) to remove unattached viruses, were incubated in maintenance medium and were maintained (2% FBS) at 37°C for 48 h. In order to improve the infection efficiency, we added 5μg/ml polyethylene (Solarbio; H8761) to the PFV virus solution when infecting HEK293T cells, and other conditions remained unchanged.

### Plasmids and Transient Transfection

The cDNA encoding TBC1D16 and its truncated plasmids were cloned into pCMV-Flag and cDNA encoding Rab5C was cloned into pCMV-HA. Further, TBC1D16-R431A, TBC1D16-R494A, TBC1D16-Q531A, TBC1D16-R431AR494A, TBC1D16-R431AQ531A, TBC1D16-R494AQ531A and TBC1D16-R431AR494AQ531A mutant plasmids were constructed based on the TBC1D16 overexpressing plasmid and were cloned into pCMV-Flag. The primers are shown in **Table S1**. The infectious pHSRV13 provirus DNA was a gift from Professor Rolf M. Flügel (German Cancer Research Center) (31). The LTR-Luc and IP-Luc were amplified from pHSRV13 provirus DNA and cloned into the pGL3-Basic plasmid (Promega). The HA-Tas and HA-Bet were amplified from pHSRV13 provirus DNA and cloned into the pCMV-HA plasmid. The His-Gag were amplified from pHSRV13 provirus DNA and cloned into the pCMV-His plasmid. The TK-Tas were amplified from pHSRV13 provirus DNA and cloned into the pRL-TK plasmid (Promega). The truncated-LTR firefly luciferase reporters were also amplified from pHSRV13 and cloned into the pGL3-Basic plasmid (Promega).

TBC1D16-specific shRNA and a nonsilencing shRNA (NC, used as a negative control) were purchased from Genechem Shanghai (China). Rab5C-specific shRNA and a nonsilencing shRNA (NC) were purchased from GenePharma Shanghai (China). Plasmids and shRNA transfections were performed using PEI transfection reagent in HEK293T cells, and using Neofect™ DNA transfection reagent in HT1080 cells. The THP-1 cells were activated by 100 ng/ml phorbol myristate acetate (PMA) for 72 h. For transient transfection of plasmids into phorbol myristate acetate-activated THP-1 cells, Lipofectamine 3000 reagent (Invitrogen) was used. All transfection experiments were carried out according to the manufacturer’s instructions.

### Antibodies

Rabbit anti-Rab5C (Proteintech, Cat. No. 27219-1-AP) and rabbit anti-TBC1D16 (Abcam, Cat. No. ab104407) were used at a dilution of 1:1000 for Western blotting. The anti-β-actin (Abcam, Cat. No. ab3280) were purchased from Abcam and used at a dilution of 1:10000 for Western blotting. Mouse anti-His (Proteintech, Cat. No. 66005-1-lg), mouse anti-Myc (Cell Signaling Technology, Cat. No. 2276S), rabbit anti-FLAG (Cell Signaling Technology, Cat. No. 14793S), mouse anti-FLAG (Cell Signaling Technology, Cat. No. 8146S) and rabbit anti-HA (Cell Signaling Technology, Cat. No. 3724S) were used at a dilution of 1:50 for immunoprecipitation. Antibody against PFV Gag was generously provided by Professor Li Zhi. The anti-Tas was produced by immunizing mice with prokaryotically expressed Tas, followed by purification according to standard procedures (18). HRP-conjugated goat anti-mouse or HRP-conjugated goat anti-rabbit secondary antibodies were from Bioprimacy and used at 1:10,000.

### Reverse Transcription and Quantitative Real-Time PCR

Quantitative real-time PCR (qPCR) has become the method of choice for determining the relative quantities of RNA (cDNA) and DNA in gene expression analysis (32). Total cellular RNA was extracted from the different treatments of cells using TRIzol reagent (Invitrogen, Carlsbad, CA, USA) according to the manufacturer’s protocol. Then cDNA was reverse transcribed from 2 μg of the total RNA using the RevertAid™ First Strand cDNA Synthesis Kit (Thermo Scientific). The mRNA level was evaluated by qRT-PCR using the SYBR green Real-Time PCR Master Mix kit (Toyobo) and was analyzed on a CFX96 sequence detection system (BIO-RAD) according to the manufacturer’s protocol. The program set on the CFX96 sequence detection system (BIO-RAD) was 95°C for 30 s, followed by 40 cycles at 95°C for 15 s, 58°C for 20 s, and 72°C for 15 s. All gene expression was normalized to the housekeeping gene β-actin, which was used as an internal standard. All quantitative real-time PCR primers are listed in **Table S2**. The relative quantitative values were calculated by the 2^-△△Ct^ method. Melting curve analysis was performed to verify the specificity of the primers, and quantification of β-actin transcripts was used to normalize RNA amounts. Statistical significance was analyzed with Student’s *t*-test (*p< 0.05, **p< 0.01, ***p< 0.001). All data are representative of three independent experiments with triplicate samples.

### Luciferase Assay

We seeded HEK293T cells into 24-well plates, and 24 h later the cells transfected with pGL3-PFV-LTR-luc or pGL3-PFV-IP-luc reporter plasmids and all of the plates were transfected with the Renilla luciferase reporter plasmid (pRL-TK, Promega) as an internal control. 48 h after transfection, passive lysis buffer (100 μl/plate) was added to the cells and dissolved for 20 min at room temperature. Then, firefly and Renilla luciferase activities were obtained using the Dual-Luciferase Reporter Assay System following the manufacturer’s protocol (Promega). All experiments were performed in triplicate. The firefly luciferase activity was normalized to the Renilla luciferase activity and expressed as the fold change relative to the activity in the vector-transfected cells. Data represent the average of three independent experiments, and error bars represent SD. Statistical significance was analyzed with Student’s *t*-test (*p< 0.05, **p< 0.01, ***p< 0.001).

### Foamy Virus Activated Luciferase (FAL) Assay

PFV indicator cell line (BHK21-derived indicator cells encoding a luciferase gene driven by the PFV LTR promoter) was donated by Professor Li Zhi (Xi’an Normal University) (17). Since the activity of LTR is strictly dependent on Tas, the luciferase gene is only expressed when Tas is present in the system, and the expression level is directly proportional to the amount of Tas. Therefore, it can be used to measure virus titer of PFV and is more sensitive than TCID50 (33). For relative viral load detection, the PFV-infected HEK293T cells (1 × 10^4^) were incubated with PFV indicator cell line (1 × 10^5^) for 48 h. Twelve hours before incubating with the infected HEK293T cells, the PFV indicator cell line was transfected with RL-TK plasmid expressing Renilla luciferase as an internal control. After 48 h, the luciferase activity was assessed.

### Western Blotting

Following transfection or infection for the indicated time, HEK293T or HT1080 cells were washed twice with ice-cold 1 × phosphate buffered saline (PBS) and lysed on ice with RIPA lysis buffer (Beyotime Biotechnology #P0013K) containing 1 × phenylmethylsulfonyl fluoride (PMSF, as a protease inhibitor). The protein concentration was quantified by BCA protein assay (Beyotime Biotechnology). Then, the protein lysates with different treatments were centrifuged at 12,000 rpm for 15 min at 4°C to remove the precipitate. The treated samples with 1 × loading buffer (5% SDS, 10% glycerol, 60 mM Tris pH 6.8, 5% β-mercaptoethanol, and 0.01% bromophenol blue) were boiled at 100°C for 10-15 min before electrophoretic separation. The protein samples were separated by 10% sodium dodecyl sulfate-polyacrylamide gel electrophoresis (SDS-PAGE) according to the different molecular weights. Then the protein samples in polyacrylamide gel were transferred to polyvinylidene fluoride (PVDF) membranes (Roche), and the membranes were blocked in 5% nonfat milk-TBST for 3 h at room temperature. After blocking, the membranes were washed with TBST for 1 min and then incubated with primary antibodies overnight at 4°C. Next, we washed the membranes with 1 × TBST for 15 min × 3 times to remove the residual primary antibodies, and then the membranes were hybridized with horseradish peroxidase (HRP)-conjugated secondary antibody (PMK Biotechnology Co., Ltd.) for 2 h at room temperature. Antibody-antigen complexes were observed using an enhanced chemiluminescence (ECL) system (Advansta, Menlo Park, CA, USA) with an ECL chemiluminescence imaging system (Tanon-5200). All data are representative of three independent experiments with triplicate samples.

### Co-IP

The cells were lysed in Nonidet P-40 lysis buffer containing 150 mM NaCl, 1 mM EDTA, 1% Nonidet P-40, and 1% protease and phosphatase inhibitor cocktail (Beyotime Biotechnology). The whole-cell lysates were pretreated with Protein A/G Plus-Agarose beads for 4 h at 4°C and then immunoprecipitated with IgG (control) or the indicated antibodies. Protein A/G Plus-Agarose (Santa Cruz Biotechnology, sc-2003) and the precipitants were washed three times with a high salt lysis buffer containing 500 mM NaCl, followed by immunoblot analysis. The antibodies were diluted in 3% to 5% (wt/vol) fat-free milk (BD Biosciences) or 3% BSA (Sigma) in TBS (1:500-1:2000).

### ChIP

ChIP assays were performed as previously described by Wu’s lab (33). Briefly, cells were crosslinked with 1% formaldehyde for 10 min on ice and terminated with 125 mM glycine for 5 min at room temperature. After cross-linking, the cells were washed with precooled PBS three times, and the DNA of the cells was extracted in chip lysis buffer (50 mM Tris-HCl pH 8.0, 1% SDS, 5 mM EDTA). Then, the DNA fragment was separated to 400-600 bp by ultrasonication. The lysate was centrifuged at 4°C for 15 min, and the precipitate was removed. The ChIP dilution buffer (20 mM Tris-HCl, pH 8.0, 150 mM NaCl, 2 mM EDTA, 1% Triton X-100) was added to the supernatant (4:1 volume) and incubated with protein G beads and antibodies at 4°C overnight. The beads were washed five times with wash buffer I, wash buffer II, wash buffer III and TE (Tris EDTA, pH 8.0) buffer × 2, and DNA was eluted in ChIP elution buffer (0.1 M NaHCO3, 1% SDS and 30 μg/ml proteinase K). The elution was incubated at 65°C overnight, and DNA was extracted with a DNA purification kit (Tiangen). The purified DNA was assayed by quantitative PCR with a SYBR Green Real-Time PCR Master Mix kit (Toyobo). The primer information is listed in **Table S2**. The data shown are the mean ± standard deviation (SD) of representative experiments. The ChIP experiments were repeated three times independently and Student’s *t*-test was used for statistical analysis (*p < 0.05, **p < 0.01, ***p < 0.001).

### ELISA

THP-1 cells were seeded into 96-well plates and cultured in a CO_2_ incubator at 37°C for 24 h. Then the cells were transfected with pCMV-Flag-TBC1D16 or TBC1D16 specific shRNA. After 24 h of transfection, the THP-1 cells were infected with PFV. The infected cells were incubated at 37°C for 2 h, and then the virus supernatant was replaced with fresh medium to continue culturing for 48 h. The concentration of IFN-β in culture supernatants was measured with a human IFN-β ELISA kit (4A BIOTECH, Cat.CHE0274).

### Statistical Analysis

Data are expressed as the means ± standard deviations. Statistical analyses were performed using GraphPad Prism software (GraphPad Software, La Jolla, CA, USA) to evaluate the differences between the control and experimental groups. Significance was determined and analyzed with Student’s *t*-test (ns *p* > 0.05, *p < 0.05, **p < 0.01, ***p < 0.001). All data are representative of three independent experiments with triplicate samples. All experiments in this study were repeated at least three times.

## Results

### TBC1D16 Expression Is Down-Regulated in PFV-Infected Cells

**Figure 1:**
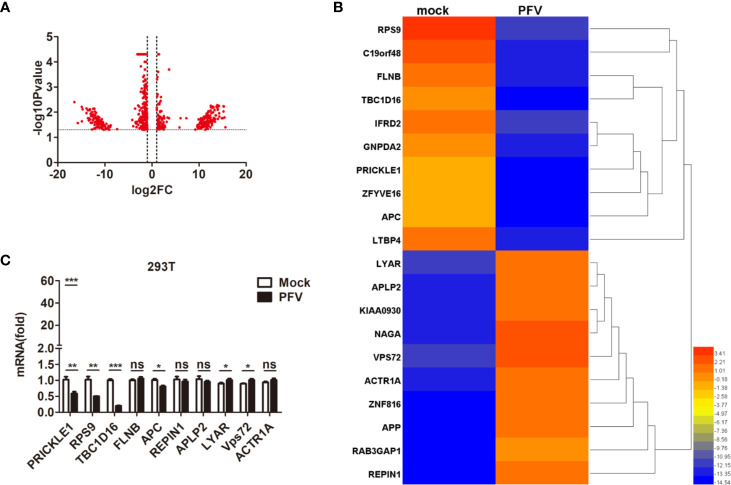
The expression of TBC1D16 is down-regulated in PFV-infected cells. **(A)** Volcano map of differentially expressed genes in the host after PFV infection. **(B)** Heat map of 20 selected genes with the most significant differences in expression. A scale from 3.41 to -14.54 represents folds of differential expression. **(C)** Expression of ten differentially expressed genes encoding functional proteins in PFV-infected HEK293T cells compared with mock-infected cells (*p < 0.05, **p < 0.01, ***p < 0.001 and ns, no significance).

In order to gain a deeper understanding of the replication strategy of PFV and its relationship with the host, RNA-seq was used to detect the expression changes of the host genes after virus infection (34). The results showed that there were 61,367 mRNAs 30,133 up-regulated mRNAs and 31,220 down-regulated mRNAs) differentially expressed in PFV-infected HEK293T cells compared to the mock-infected cells (**Figure 1A**). Twenty genes with the most significant differences in expression (10 up-regulated mRNAs and 10 down-regulated mRNAs) are shown in the heat map (**Figure 1B**). Subsequently, 10 differentially expressed genes (5 up-regulated mRNAs and 5 down-regulated mRNAs) encoding functional proteins were confirmed by qPCR assays (**Figure 1C**). These results showed that the expression of TBC1D16 was significantly down-regulated in PFV-infected HEK293T cells.

### TBC1D16 Inhibits the Replication of PFV

**Figure 2:**
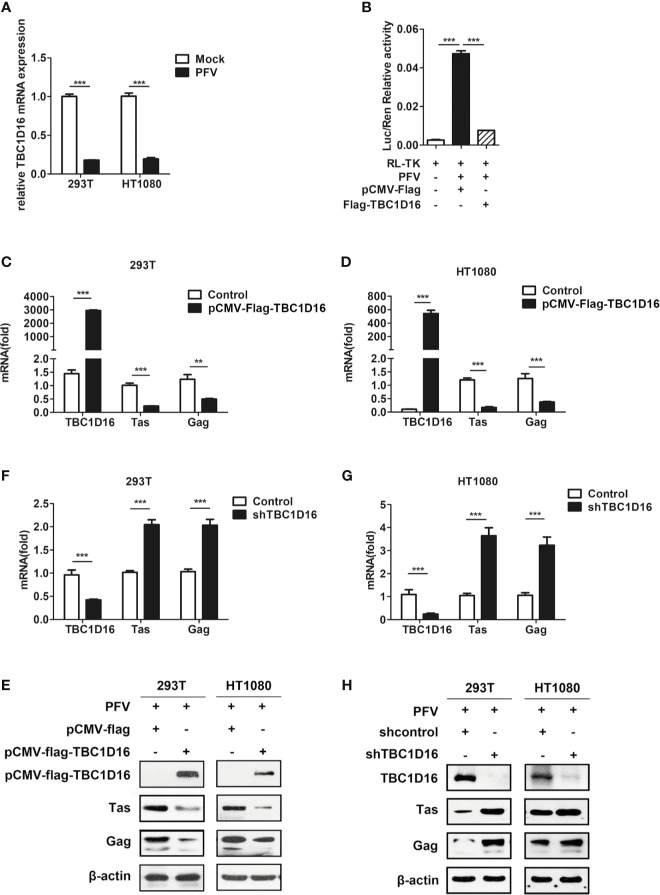
TBC1D16 inhibits the replication of PFV. **(A)** After infection with PFV (MOI = 0.5) for 48 h, the mRNA expression of TBC1D16 in HEK293T and HT1080 cells was detected by qPCR (***p < 0.001). **(B)** The relative viral load in the presence or absence of overexpressed TBC1D16 was analyzed in PFV indicator cell line. RL-TK (1.5 μg) was transfected as an internal control. **(C, D)** pCMV-Flag-TBC1D16 or pCMV-Flag (as an empty control) were transfected into HEK293T and HT1080 cells for 24 h. After transfection, the cells were infected with PFV (MOI = 0.5) for 48 h. The mRNA or protein expression of PFV Tas and Gag was detected by qPCR (**p < 0.01 and ***p < 0.001). **(E)** pCMV-Flag-TBC1D16 or pCMV-Flag (as an empty control) were transfected into HEK293T and HT1080 cells for 24 h. After transfection, the cells were infected with PFV (MOI = 0.5) for 48 h. The protein expression of PFV Tas and Gag was detected by western blotting. **(F, G)** The specific shRNA and shControl (as a negative control) were transfected into HEK293T and HT1080 cells for 24 h to knock down the expression of TBC1D16 and then were infected with PFV (MOI = 0.5) for 48 h. The mRNA expression of PFV Tas and Gag was detected by qPCR (***p < 0.001). **(H)** The specific shRNA and shControl (as a negative control) were transfected into HEK293T and HT1080 cells for 24 h to knock down the expression of TBC1D16 and then were infected with PFV (MOI = 0.5) for 48 h. The protein expression of PFV Tas and Gag was detected by western blotting.

TBC1D16 is a member of the TBC protein family. Previous studies have demonstrated that TBC1D16 was involved in various types of diseases, such as high systolic blood pressure (35), obesity (36) and cancer (37). Based on our current and previous studies, HEK293T cells and human fibrosarcoma (HT1080) cells could be suitable for *in vitro* models to study the replication strategy of PFV (38). In order to confirm whether TBC1D16 is involved in PFV replication, we detected the expression of TBC1D16 in mock-infected or PFV-infected cells by qPCR. These results showed that TBC1D16 gene expression was significantly reduced in PFV-infected HEK293T (5.6-fold change) and HT1080 cells (5.3-fold change) compared with mock-infected cells at 48 h (**Figure 2A**, *p* < 0.001). Then, we used the PFV indicator cell line to investigate the effect of TBC1D16 on PFV replication. We co-incubated the PFV-infected HEK293T cells with the PFV indicator cell line for 48 h. 12 h before the co-incubation, the RL-TK plasmid expressing Renilla luciferase was transfected into the PFV indicator cell line as an internal control (39). The results of the dual luciferase reporter gene experiment showed that HEK293T cells infected with PFV strongly activated the expression of luciferase gene in the PFV indicator cell line. Compared with the control group (pCMV-Flag), overexpression of TBC1D16 significantly suppressed the level of the PFV viral load (**Figure 2B**, *p* < 0.001). These results suggested that TBC1D16 restricted PFV replication.

In order to verify the above results, we also used qPCR and western blot to detect the effect of TBC1D16 on the expression of PFV Tas and Gag. TBC1D16 was overexpressed in HEK293T and HT1080 cells for 24 h using the pCMV-Flag-TBC1D16 plasmid, and then the cells were infected with PFV for 48 h. The results showed that mRNA expression of Tas (4.2-fold change) and Gag (1.8-fold change) was remarkably suppressed in pCMV-Flag-TBC1D16 transfected HEK293T cells compared to control vector (pCMV-Flag) treated HEK293T cells (**Figure 2C**, *p* < 0.001). We also found that TBC1D16 inhibited the transcription of PFV Tas and Gag in a dose dependent way (**Figure S1**). Similarly, Tas and Gag expression was detected in HT1080 cells, and it showed that there was a 6.9-fold decrease in Tas and a 2.6-fold decrease in Gag in the TBC1D16 overexpression group compared to the control vector (pCMV-Flag) group (**Figure 2D**, *p* < 0.001). Additionally, compared with the control group, the protein expression of Tas and Gag in the TBC1D16 overexpression group was also reduced (**Figure 2E**). In contrast, knocking down TBC1D16 with shRNA in HEK293T cells resulted in elevated levels of the mRNA expression of Tas (2-fold change) and Gag (2-fold change) compared with the shNC (negative control) group (**Figure 2F**, *p* < 0.001). The same result was found in HT1080 cells, and the mRNA expression of Tas (3.6-fold change) and Gag (3.2-fold change) was significantly increased after TBC1D16 silencing (**Figure 2G**, *p* < 0.001), while the protein expression of Tas and Gag was increased in the TBC1D16 knockdown group compared with the control group (**Figure 2H**). Collectively, these findings indicated that TBC1D16 inhibits the replication of PFV.

### TBC1D16 Affects the Tas-Dependent Transactivation of PFV LTR and IP Promoters

**Figure 3:**
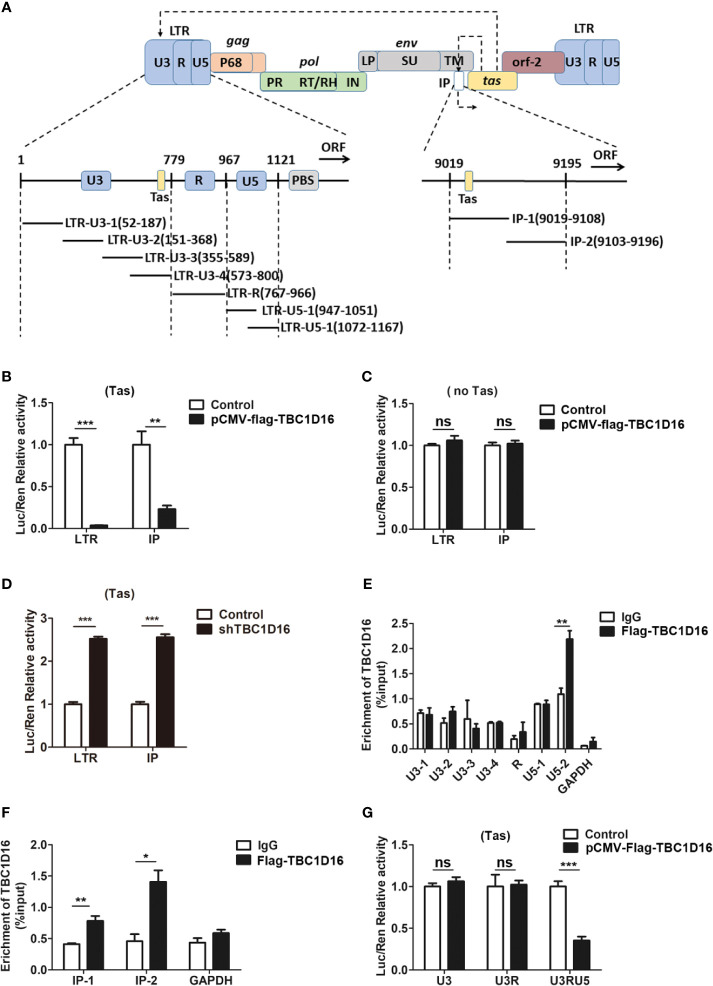
TBC1D16 affects the Tas-dependent transactivation of the PFV LTR and IP promoters. **(A)** Structural diagram of PFV LTR and IP promoter. **(B)** HEK293T cells seeded in 24-well plates were contransfected with pCMV-Flag-TBC1D16 (400 ng, and pCMV-Flag as an empty control), pRL-TK (3 ng), and pGL3-PFV-LTR-luc (30 ng) or pGL3-PFV-IP-luc (20 ng) firefly luciferase reporter. These cotransfected plasmids were combined with pTK-Tas (30 ng for pGL3-PFV-LTR-luc or 20 ng for pGL3-PFV-IP-luc). A luciferase reporter assay was used to detect LTR and IP promoter activity (Renilla luciferase as an internal control) (**p < 0.01, ***p < 0.001). **(C)** Luciferase assays were performed similarly as in **(B)**. But these cotransfected plasmids were combined without pTK-Tas (*ns*, no significance). **(D)** The specific shRNA of TBC1D16 and shControl (as a negative control), pRL-TK (3 ng), pTK-Tas (30 ng for pGL3-PFV-LTR-luc or 20 ng for pGL3-PFV-IP-luc) and pGL3-PFV-LTR-luc (30 ng) or pGL3-PFV-IP-luc (20 ng) firefly luciferase reporter were transfected into HEK293T cells for 48 h. Luciferase reporter assay was used to detect the LTR and IP promoter activity (Renilla luciferase as an internal control) (***p < 0.001). **(E, F)** pCMV-Flag-TBC1D16 (3 μg) and pGL3-PFV-LTR-luc (3 μg) or pGL3-PFV-IP-luc (3 μg) were cotransfected into HEK293T cells for 48 h, and ChIP assay was used to detect the enrichment of TBC1D16 on the PFV LTR and IP promoter. ChIP-qPCR data were normalized by the percent input method (%input with IgG as control). The data are presented as the means ± SD (*p < 0.05 and **p < 0.01). **(G)** pCMV-Flag-TBC1D16 (400 ng and pCMV-Flag as an empty control), pRL-TK (3 ng), pTK-Tas (30 ng) and truncated pGL3-PFV-LTR-luc (30 ng) firefly luciferase reporter were cotransfected into HEK293T cells for 48 h. Luciferase reporter assay was used to detect the truncated LTR promoter activity (Renilla luciferase as an internal control) (***p < 0.001 and *ns*, no significance).

LTR and IP promoters are key to regulating PFV replication and determining whether the infection is lytic or persistent (40). In the initial stage of PFV replication, IP has modest basal activity without Tas, which can drive initial Tas expression, and then Tas transactivates LTR and IP promoters to initiate virus replication (41). To further study the role of TBC1D16 in inhibiting PFV replication, we determined whether TBC1D16 suppressed the activity of LTR or IP promoters by luciferase assay. In this reporter assay system, Renilla luciferase was selected as an internal control to minimize or possibly even eliminate the experimental variability (42). In our study, HEK293T cells were seeded into 24-well plates and transfected with pCMV-Flag-TBC1D16 and pTK-Tas expression plasmids together with LTR-Luc (pGL3-PFV-LTR-luc) or IP-Luc (pGL3-PFV-IP-luc) reporter plasmid for 48 h. These results showed that the overexpression of TBC1D16 inhibited the Tas-dependent transactivation of PFV LTR and IP promoter, and the activation levels of LTR (26.5-fold, *p* < 0.001) and IP (4.3-fold, *p* < 0.01) in the TBC1D16 overexpression group were significantly reduced compared to the control vector (pCMV-Flag) group (**Figure 3B**). In contrast, TBC1D16 had no effect on LTR or IP activation when Tas was absent (**Figure 3C**), while knocking down TBC1D16 with shRNA enhanced LTR and IP promoter activity, and the activation levels of LTR (2.5-fold) and IP (2.6-fold) were significantly increased in the TBC1D16 silenced group compared to the shNC (negative control) group (**Figure 3D**, *p* < 0.001).

It is universally known that the chromatin immunoprecipitation (ChIP) assay is an effective method to study protein-gene interactions *in vivo* (43). To determine the enriched fragments of TBC1D16 on PFV LTR and IP, we designed 6 pairs of primers for PFV LTR and 2 pairs of primers for PFV IP to confirm the enrichment sites of TBC1D16 on LTR and IP by ChIP assay (**Figure 3A**). The pCMV-Flag-TBC1D16 and pGL3-PFV-LTR-luc overexpressed plasmids were cotransfected into HEK293T cells for 48 h, and the DNA interacting with TBC1D16 was purified by anti-FLAG antibody and detected by qPCR. Interestingly, we found that TBC1D16 was significantly enriched in the LTR U5-2 section (2-fold change, *p* < 0.01) and both sections of IP (1.9-fold change of IP-1 (*p* < 0.01) and 3.1-fold change of IP-2 (*p* < 0.05)) compared with the control (IgG) group (**Figures 3E, F**). The ChIP experiments were repeated 3 times independently. In addition, this experiment was further validated by luciferase assay. The LTR truncated reporter plasmids and pCMV-Flag-TBC1D16 overexpressing plasmid were cotransfected into HEK293T cells for 48 h. The luciferase assay results showed that, in the TBC1D16 overexpression group the activation level of the LTR truncated reporter plasmid containing U5 (2.8-fold change) was significantly decreased compared with that of the control vector (pCMV-Flag) group (**Figure 3G**, *p* < 0.001). These data suggest that TBC1D16 not only restrains Tas-dependent transactivation of PFV LTR and IP promoters but also enriches the U5 region of LTR and two regions of the IP promoter.

### TBC Domain Is Important for TBC1D16 to Inhibit PFV Replication

**Figure 4:**
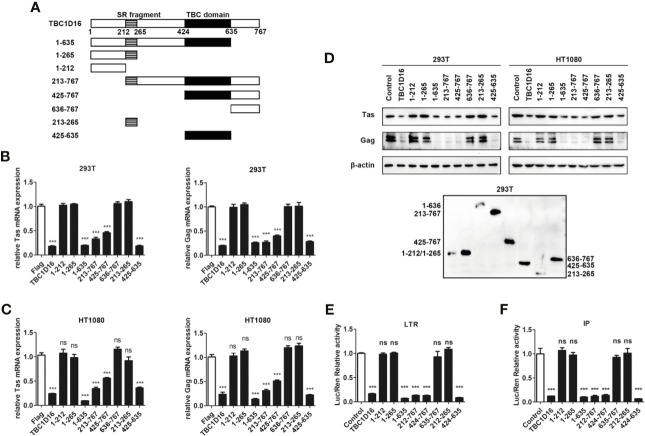
The TBC domain of TBC1D16 is important for TBC1D16 to inhibit PFV transcription and replication. **(A)** Schematic diagram of full-length TBC1D16, indicating the C-terminal TBC domain and Ser rich (SR) domain,and truncation mutants used in subsequent analyses. **(B, C)** pCMV-Flag-TBC1D16 truncated plasmids (pCMV-Flag as an empty control) were transfected into HEK293T **(B)** and HT1080 **(C)** cells for 24 h. After transfection, cells were infected with PFV (MOI = 0.5) for 48 h. The mRNA expression of PFV Tas and Gag was detected by qPCR (***p < 0.001 and *ns*, no significance). **(D)** pCMV-Flag-TBC1D16 truncated plasmids (pCMV-Flag as an empty control) were transfected into HEK293T and HT1080 cells for 24 h. After transfection, cells were infected with PFV (MOI = 0.5) for 48 h. The protein expression of PFV Tas and Gag was detected by western blotting. The expression of TBC1D16 truncated plasmids were detected by anti-Flag antibody in HEK293T cells. **(E)** pCMV-Flag-TBC1D16 truncated plasmids (400 ng and pCMV-Flag as an empty control), pRL-TK (3 ng), pTK-Tas (30 ng) and pGL3-PFV-LTR-luc (30 ng) firefly luciferase reporter plasmid were cotransfected into HEK293T cells for 48 h. Luciferase reporter assay was used to detect LTR promoter activity (Renilla luciferase was used as an internal control) (***p < 0.001 and *ns*, no significance). **(F)** pCMV-Flag-TBC1D16 truncated plasmids (400 ng and pCMV-Flag as an empty control), pRL-TK (3 ng), pTK-Tas (20 ng) and pGL3-PFV-IP-luc (20 ng) firefly luciferase reporter plasmid were cotransfected into HEK293T cells for 48 h. Luciferase reporter assay was used to detect IP promoter activity (Renilla luciferase as an internal control) (***p < 0.001 and *ns*, no significance).

The TBC domain is the conserved domain of the TBC protein family (44), and TBC1D16 also has a conserved serine-enriched (SR) fragment at the C-terminus (**Figure 4A**). To determine whether these two fragments can work separately and to compare which fragment is more important for the function of TBC1D16, we constructed a series of truncated plasmids that contain the TBC domain or the serine-enriched fragment (**Figure 4A**). After transfection with the different truncated plasmids for 24 h the HEK293T and HT1080 cells were infected with PFV for 48 h, and then the expression of Tas and Gag was detected by qPCR and western blot analyses. These results showed that the mRNA expression of Tas and Gag in HEK293T and HT1080 cells was significantly decreased in the group with truncated plasmids containing the TBC domain compared with the control vector (pCMV-Flag) group (**Figures 4B, C**, *p* < 0.001). Western blot experiments detected the same results in HT1080 and HEK293T cells (**Figure 4D**), and the expression of TBC1D16 truncated plasmids was also detected by anti-Flag antibody in HEK293T cells (**Figure 4D**). The activation of two promoters of LTR and IP was significantly decreased in the group with truncated plasmids containing the TBC domain compared with the control vector (pCMV-Flag) group, while the SR fragment had no effect (**Figures 4E, F**, *p* < 0.001). Considered together, these findings indicate that the TBC domain of TBC1D16 plays a key role in inhibiting the replication of PFV.

### Highly Conserved Amino Acid Residues R494 and Q531 in the TBC Domain Are Important for TBC1D16 to Inhibit PFV Replication

**Figure 5:**
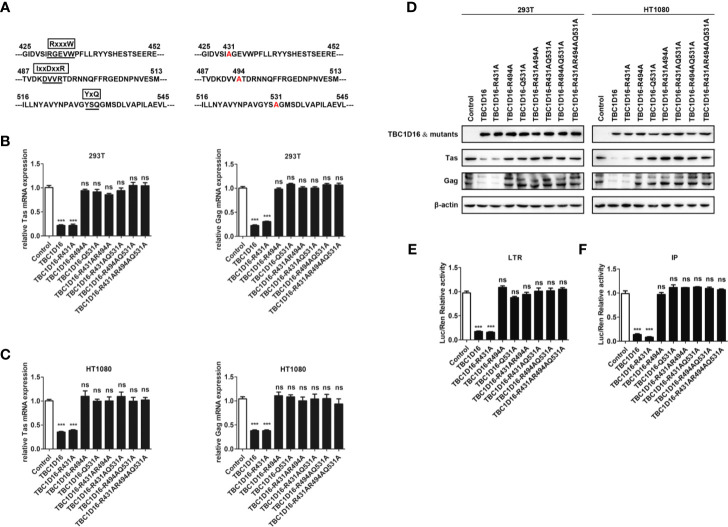
Highly conserved amino-acid residues R494 and Q531 in the TBC domain are important for TBC1D16 to inhibit PFV replication. **(A)** Schematic diagram of the key locus in the TBC domain of TBC1D16 indicating three signature motifs, which were specific locus mutants used in subsequent analyses. **(B, C)** pCMV-Flag-TBC1D16 specific locus mutant plasmids (pCMV-Flag as an empty control) were transfected into HEK293T and HT1080 cells for 24 h. After transfection, cells were infected with PFV (MOI = 0.5) for 48 h. The mRNA expression of PFV Tas and Gag was detected by qPCR (***p < 0.001 and *ns* no significance). **(D)** pCMV-Flag-TBC1D16 specific locus mutant plasmids (pCMV-Flag as an empty control) were transfected into HEK293T and HT1080 cells for 24 h. After transfection, cells were infected with PFV (MOI = 0.5) for 48 h. The protein expression of PFV Tas and Gag was detected by western blot. **(E, F)** pCMV-Flag-TBC1D16 specific locus mutant plasmids (400 ng and pCMV-Flag as an empty control), pRL-TK (3 ng), pTK-Tas (30 ng for pGL3-PFV-LTR-luc or 20 ng for pGL3-PFV-IP-luc) and pGL3-PFV-LTR-luc (30 ng) or pGL3-PFV-IP-luc (20 ng) firefly luciferase reporter plasmid were cotransfected into HEK293T cells for 48 h. Luciferase reporter assay was used to detect LTR and IP promoter activity (Renilla luciferase as an internal control) (***p < 0.001).

Most TBC proteins accelerate GTP hydrolysis by a similar dual-finger mechanism including the “arginine finger” and “glutamine finger” (45). In the TBC domain, there are three conserved motifs – RxxxW, IxxDxxR and YxQ – and the residues from the IxxDxxR motif and the YxQ motif are crucial for GTP hydrolysis (46). To verify whether arginine and glutamine from three signature motifs (RxxxW, IxxDxxR and YxQ) in the TBC domain of TBC1D16 are also critical to inhibit PFV replication, we constructed a TBC1D16 overexpressing plasmid with the key site mutations, including single site mutant plasmids, double site mutant plasmids and triple site mutant plasmids (**Figure 5A**). The double site mutant plasmids were obtained by mutating two arbitrary sites in the three signature motifs (RxxxW, IxxDxxR and YxQ) of the TBC domain. The results of qPCR and western blot showed that the expression of Tas and Gag in HEK293T and HT1080 cells were not changed in the mutant plasmid group containing R494A or Q531A, compared with the control vector (pCMV-Flag) group (**Figures 5B–D**). Moreover, Tas-dependent transactivation of PFV LTR and IP promoter was also unchanged in the different mutant plasmid groups containing R494A or Q531A compared with the control vector (pCMV-Flag) group (**Figures 5E, F**). Notably, in the TBC1D16-R431A mutant plasmid-transfected group, the expression of Tas (4.6-fold change in HEK293T cells and 2.6-fold change in HT1080 cells) and Gag (3.3-fold change in HEK293T cells and 2.8-fold change in HT1080 cells) was significantly reduced compared to the control vector (pCMV-Flag) group (**Figures 5B, C**, *p* < 0.001). And the activity of LTR (6.2-fold change) and IP (11.9-fold change) promoters was significantly reduced compared to those of the control vector (pCMV-Flag) group (**Figures 5E F**, *p* < 0.001). These results suggested that R494 and Q531 in the TBC domain are important for TBC1D16 to inhibit PFV replication.

We also detected the enrichment of different TBC1D16 mutant plasmids on the PFV LTR and IP promoters. TBC1D16 with the R431 mutation was similar to wild-type TBC1D16, which were enriched at the U5 region of the LTR and two regions of the IP promoter compared to the control (IgG) group (**Figure S2**, **p < 0.01 and ***p < 0.001). However, TBC1D16 with R494A or Q531A could not be enriched at the LTR and IP promoters (**Figure S2**). Taken together, these results demonstrated that the TBC domain is important for the function of TBC1D16 and that the conserved amino acid residues R494 and Q531 in the TBC domain of TBC1D16 are two critical amino acid sites for antiviral activity.

### Rab5C Rather Than Rab4A Inhibits the Replication of PFV

Rab GTPases behave as membrane-associated molecular switches that can be tightly controlled by GDP-GTP exchange, as well as the cycle between GTP (guanosine triphosphate)-bound “ON” and GDP (guanosine diphosphate)-bound “OFF” conformational states (47). The cycle is controlled by two regulatory enzymes: specific guanine nucleotide exchange factors (GEFs)-catalyze the exchange GDP for GTP in Rab GTPases (48), and GTPase-activating proteins (GAPs) facilitate GTP hydrolysis (49). TBC proteins have been shown to function as Rab-specific GTPase-activating proteins (TBC/RABGAPs) and act as negative regulators of Rab GTPases (50). As the GAP of Rab4A, TBC1D16 regulates the circulation of transferrin receptor and EGFR trafficking and signaling (51). Additionally, TBC1D16-47KD, a short isoform of TBC1D16, can reduce EGFR activity in metastatic cells by targeting Rab5C (37).

**Figure 6:**
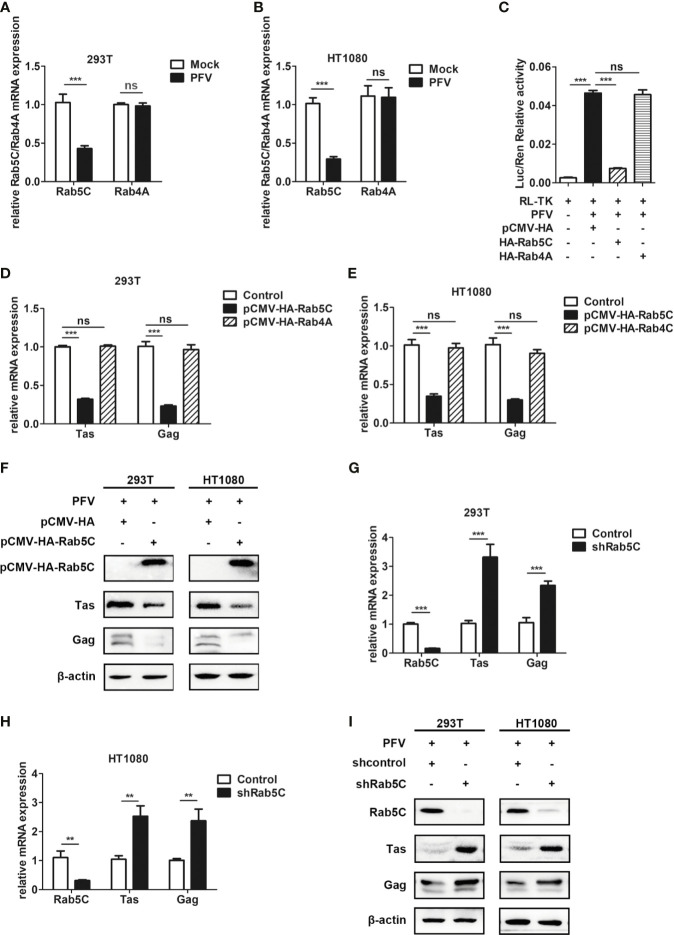
Rab5C, rather than Rab4A, inhibits the replication of PFV. **(A, B)** Gene expression of Rab5C and Rab4A after PFV infection. After infection with PFV for 48 h, the mRNA expression of Rab5C and Rab4A in HEK293T and HT1080 cells was detected by qPCR (***p < 0.001 and *ns*, no significance). **(C)** The relative viral load in the presence or absence of overexpressed Rab5C or Rab4A was analyzed in PFV indicator cell line. RL-TK (1.5 μg) was transfected as an internal control. **(D, E)** pCMV-HA-Rab5C or pCMV-HA-Rab4A and pCMV-HA (as an empty control) were transfected into HEK293T and HT1080 cells for 24 h. After transfection, cells were infected with PFV (MOI = 0.5) for 48 h. The mRNA expression of PFV Tas and Gag was detected by qPCR (***p < 0.001). **(F)** pCMV-HA-Rab5C and pCMV-HA (as an empty control) were transfected into HEK293T and HT1080 cells for 24h. After transfection, cells were infected with PFV (MOI = 0.5) for 48 h. The protein expression of Tas and Gag were detected by western blotting. **(G, H)** The specific shRNA of Rab5C or shControl (as a negative control) was transfected into HEK293T and HT1080 cells for 24 h to knockdown the expression of Rab5C and then the cells were infected with PFV (MOI = 0.5) for 48 h. The mRNA expression of PFV Tas and Gag was detected by qPCR (**p < 0.01 and ***p < 0.001). **(I)** The specific shRNA of Rab5C or shControl (as a negative control) was transfected into HEK293T and HT1080 cells for 24 h to knock down the expression of Rab5C, and then the cells were infected with PFV (MOI = 0.5) for 48 h. The protein expression of Tas and Gag were detected by western blotting.

To verify whether Rab5C or Rab4A plays a role in TBC1D16 inhibiting PFV replication, we constructed Rab5C and Rab4A overexpressing plasmids with HA labeling. We found that the expression of Rab5C was significantly decreased in HEK293T cells (2.4-fold change) and HT1080 cells (3.5-fold change) in the PFV-infected group compared to the mock-infected group, while Rab4A was not affected in HEK293T and HT1080 cells (**Figures 6A, B**, *p* < 0.001). In order to test whether Rab5C affected PFV replication, we used the PFV reporter cell line to detect the effect of Rab5C on PFV viral load. The RL-TK plasmid was transfected into the PFV indicator cell line for 12 h. Then the PFV-infected HEK293T cells were incubated with the PFV indicator cell line for 48 h. The results of the luciferase reporter experiment showed that overexpression of Rab5C instead of Rab4A significantly reduced the expression of the luciferase gene caused by PFV infection in the PFV reporter cell line (**Figure 6C**, *p* < 0.001). We also detected the effect of Rab5C on PFV replication by detecting the expression of PFV Tas and Gag. The Rab5C and Rab4A were overexpressed in HEK293T and HT1080 cells for 24 h, and then these cells were infected with PFV for 48 h. The qPCR results showed that the mRNA expression levels of Tas (3.1-fold change) and Gag (4.4-fold change) in Rab5C-transfected HEK293T cells were significantly reduced compared to those in the control vector (pCMV-HA) group (**Figure 6D**, *p* < 0.001). In HT1080 cells, the mRNA expression levels of Tas (2.9-fold) and Gag (3.4-fold) were also reduced in the Rab5C group compared to the control vector (pCMV-HA) group (**Figure 6E**, *p* < 0.001). However, the expression of Tas and Gag in Rab4A transfected cells did not change significantly compared with the control vector (pCMV-HA) group (**Figures 6D, E**, *p* < 0.001). The protein expression of Tas and Gag in HEK293T and HT1080 cells was also detected by western bolt analysis, and it was significantly down-regulated compared to the controls (**Figure 6F**). In contrast, knocking down Rab5C by shRNA increased the expression of Tas (3.3-fold change) and Gag (2.3-fold change) in HEK293T cells compared to the shNC (negative control) group (**Figure 6G**, *p* < 0.001). The same result was detected in HT1080 cells, and the mRNA expression of Tas (2.5-fold change) and Gag (2.3-fold change) was significantly increased after Rab5C silencing (**Figure 6H**, *p* < 0.01). We also detected the protein expression of Tas and Gag in HEK293T and HT1080 cells, and found a significant increase compared to the controls (**Figure 6I**). These findings indicate that Rab5C, not Rab4A, played a critical role in inhibiting the replication of PFV.

### QESTIGAAF (Amino Acid Residues 50-58) Is Important for Rab5C to Inhibit PFV Replication

**Figure 7:**
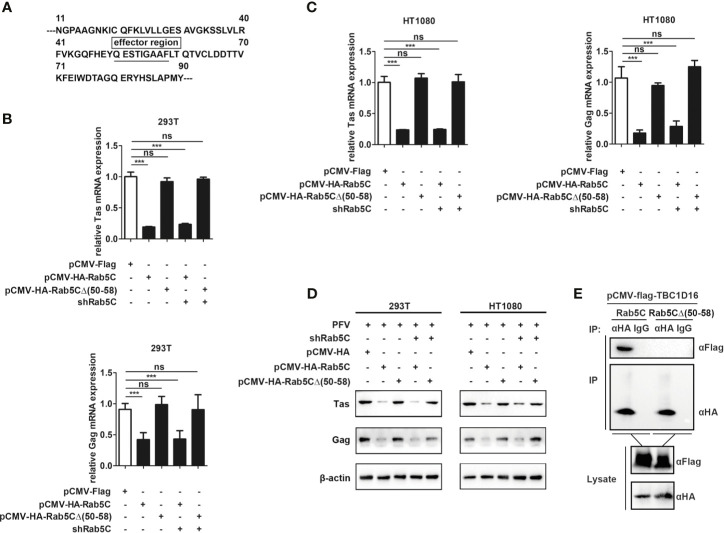
QESTIGAAF (amino-acid residues 50-58) is important for Rab5C to inhibit PFV replication. **(A)** Schematic diagram of the effector region (amino-acid residues 50-58) of Rab5C. **(B–D)** QESTIGAAF (amino-acid residues 50-58) is important for Rab5C to inhibit PFV replication. **(B, C)** pCMV-HA-Rab5C or pCMV-HA-Rab5CΔ(50-58) (400 ng and pCMV-HA as an empty control) were transfected into HEK293T and HT1080 cells for 24 h. In another group, pCMV-HA-Rab5C or pCMV-HA-Rab5CΔ(50-58) were cotransfected with specific shRNA of Rab5C into HEK293T and HT1080 cells for 24 h. After transfection, cells were infected with PFV (MOI = 0.5) for 48 h. The mRNA expression of Tas and Gag were detected by qPCR (***p < 0.001 and *ns*, no significance). **(D)** pCMV-HA-Rab5C or pCMV-HA-Rab5CΔ(50-58) (400 ng and pCMV-HA as an empty control) were transfected into HEK293T and HT1080 cells for 24 h. In another group, pCMV-HA-Rab5C or pCMV-HA-Rab5CΔ(50-58) were cotransfected with specific shRNA of Rab5C into HEK293T cells for 24 h. After transfection, cells were infected with PFV (MOI = 0.5) for 48 h. The protein expression of Tas and Gag was detected by Western blotting. **(E)** QESTIGAAF (amino-acid residues 50-58) is important for the interaction between Rab5C and TBC1D16. pCMV-Flag-TBC1D16 (3 μg) and pCMV-HA-Rab5C (3 μg) or pCMV-HA-Rab5CΔ(50-58) (3 μg) were cotransfected into HEK293T cells for 48 h, then Co-immunoprecipitation and immunoblot analysis were used to detect the interaction between TBC1D16 and Rab5C or Rab5CΔ(50-58).

To further explore the specific mechanism of Rab5C in inhibiting PFV replication, we checked the structure of Rab5C on the UniProt platform (https://www.uniprot.org/) and found that there was an effector region containing 9 amino acids in Rab5C (**Figure 7A**). We constructed a Rab5C mutant plasmid with an HA tag (pCMV-HA-Rab5CΔ (50–58)) that deleted the effector region (QESTIGAAF) to determine whether the effector region was important for the function of Rab5C. The pCMV-HA-Rab5CΔ (50–58) plasmid was transfected into HEK293T and HT1080 cells for 24 h, and then these cells were infected with PFV for 48 h. These results showed that the mRNA expression of Tas (5.4-fold change) and Gag (2.4-fold change) was significantly reduced in Rab5C overexpression HEK293T cells compared with the control vector (pCMV-HA) cells, while there was no difference in the cells transected with Rab5CΔ (50–58) (**Figure 7B**, *p* < 0.001). The mRNA expression levels of Tas (4.2-fold change) and Gag (5.1-fold change) in HT1080 cells were also reduced compared to the control vector (pCMV-HA) group (**Figure 7C**, *p* < 0.001). Western blot analysis showed that overexpression of pCMV-HA-Rab5CΔ (50–58) did not affect the protein expression of Tas and Gag compared to the control group (**Figure 7D**). In addition, rescue experiments showed that exogenously transfected Rab5C could significantly reduce the mRNA and protein expression of Tas and Gag after silencing Rab5C with shRNA, while Rab5CΔ (50–58) had no effect on Tas and Gag expression. (**Figures 7B–D**, *p* < 0.001). The results of Co-IP showed that there existed an interaction between Rab5C and TBC1C16, while no interaction was detected between Rab5CΔ (50–58) and TBC1D16 (**Figure 7E**). These results suggest that the effector region QESTIGAAF (amino-acid residues 50-58) of Rab5C is pivotal for inhibiting PFV replication.

### The Interaction Between TBC1D16 and Rab5C Is Essential to Inhibit PFV Replication

**Figure 8:**
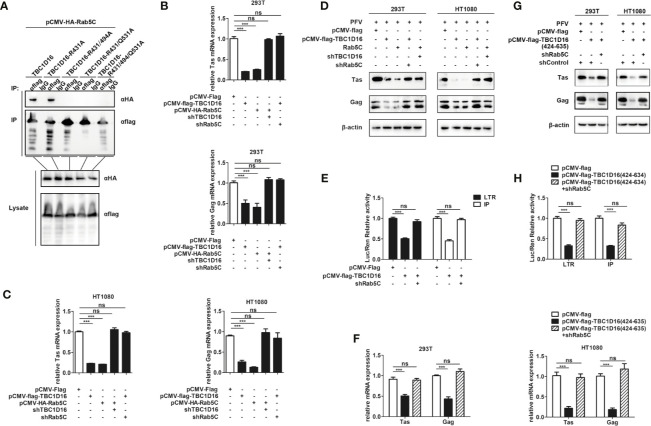
The interaction between TBC1D16 and Rab5C is essential to inhibit PFV replication. **(A)** The interaction of Rab5C and TBC1D16. pCMV-Flag-TBC1D16 specific locus mutant plasmids (3 μg) and pCMV-HA-Rab5C (3 μg) were cotransfected into HEK293T cells for 48 h. Co-immunoprecipitation and immunoblot analysis were used to detect the interaction between TBC1D16 with different locus mutants and Rab5C in HEK293T cells. **(B, C)** pCMV-Flag-TBC1D16 or pCMV-HA-Rab5C and pCMV-Flag (as an empty control) were transfected into HEK293T and HT1080 cells for 24 h. In another group, pCMV-HA-Rab5C and specific shRNA of TBC1D16 or pCMV-Flag-TBC1D16 and specific shRNA of Rab5C were cotransfected into HEK293T and HT1080 cells for 24 h. After transfection, cells were infected with PFV (MOI = 0.5) for 48 h. The mRNA expression of Tas and Gag was detected by qPCR (***p < 0.001 and *ns*, no significance). **(D)** pCMV-Flag-TBC1D16 or pCMV-HA-Rab5C and pCMV-Flag (as an empty control) were transfected into HEK293T and HT1080 cells for 24 h. In another group, pCMV-HA-Rab5C and specific shRNA of TBC1D16 or pCMV-Flag-TBC1D16 and specific shRNA of Rab5C were cotransfected into HEK293T and HT1080 cells for 24 h. After transfection, cells were infected with PFV (MOI = 0.5) for 48 h. The protein expression of Tas and Gag was detected by western blot analysis. **(E)** pCMV-Flag-TBC1D16 (400 ng), pRL-TK (3 ng), pTK-Tas (30 ng for pGL3-PFV-LTR-luc or 20 ng for pGL3-PFV-IP-luc) and pGL3-PFV-LTR-luc (30 ng) or pGL3-PFV-IP-luc (20 ng) firefly luciferase reporter plasmid were cotransfected with specific shRNA of Rab5C into HEK293T cells for 48 h. Luciferase reporter assay was used to detect the LTR and IP promoter activity (Renilla luciferase as an internal control) (***p < 0.001 and *ns*, no significance). **(F, G)** pCMV-Flag-TBC1D16 (424-635) and pCMV-Flag (as an empty control) were transfected into HEK293T and HT1080 cells for 24 h. These cotransfected plasmids were combined with or without specific shRNA of Rab5C. After transfection, cells were infected with PFV (MOI = 0.5) for 48 h. The mRNA or protein expression of PFV Tas and Gag was detected by qPCR and western blotting (***p < 0.001 and *ns*, no significance). **(H)** pCMV-Flag-TBC1D16 (424-635) (400 ng), pRL-TK (3 ng), pTK-Tas (30 ng for pGL3-PFV-LTR-luc or 20 ng for pGL3-PFV-IP-luc) and pGL3-PFV-LTR-luc (30 ng) or pGL3-PFV-IP-luc (20 ng) firefly luciferase reporter were cotransfected with specific shRNA of Rab5C into HEK293T cells for 48 h. Luciferase reporter assay was used to detect the LTR and IP promoter activity (Renilla luciferase as an internal control) (***p < 0.001 and *ns*, no significance).

To explore the different roles of TBC1D16 and Rab5C in inhibiting PFV replication, we investigated the interactions between Rab5C and various mutants of TBC1D16. The Co-IP results showed that 5C can interact with TBC1D16 or TBC1D16-R431A, but there was no interaction between Rab5C and TBC1D16-R494A or TBC1D16-Q531A (**Figure 8A**). These results confirmed that R494 and Q531 of the TBC domain in TBC1D16 were highly important for the interaction between them. Next, we further investigated whether TBC1D16 and Rab5C can inhibit PFV replication independently. TBC1D16-overexpressing plasmid and Rab5C specific shRNA (or Rab5C-overexpressing plasmid and TBC1D16 specific shRNA) were simultaneously transfected into HEK293T and HT1080 cells for 24 h, and then these cells were infected with PFV for 48 h. We found that overexpression of TBC1D16 or Rab5C reduced the mRNA and protein expression of Tas and Gag (**Figure 2** and **Figure 6**). With Rab5C knocking down, TBC1D16 was unable to inhibit the expression of Tas and Gag. Similarly, Rab5C was unable to inhibit the expression of Tas and Gag when TBC1D16 was silenced (**Figures 8B–D**, *p* < 0.001). The luciferase assay showed that TBC1D16 inhibited the activation of LTR and IP promoters, but in the absence of Rab5C, LTR and IP activation was not different in the TBC1D16 group compared with the control group (**Figures 8E**, *p* < 0.001). We also found that the truncated plasmids containing the TBC domain of TBC1D16 could suppress the expression of Tas and Gag (**Figure 4**). The pCMV-Flag-TBC1D16 (424-635) overexpressing plasmid was transfected into HEK293T and HT1080 cells for 24 h and then these transfected cells were infected with PFV for 48 h. The qPCR results showed that the mRNA expression of Tas (1.8-fold change) and Gag (2.3-fold change) in HEK293T cells was remarkably reduced in the pCMV-Flag-TBC1D16 (424-635) overexpressing group compared to the control vector (pCMV-Flag) group (**Figure 8F**, *p* < 0.001). The mRNA expression levels of Tas (4.6-fold change) and Gag (5.2-fold change) in HT1080 cells were also significantly reduced compared to the control vector (pCMV-HA) group (**Figure 8F**, *p* < 0.001). Compared with the control group, the protein expression of Tas and Gag in the TBC1D16 (424-635) overexpression group was also reduced (**Figure 8G**). When Rab5C was knocked down, TBC1D16 (424-635) had no effect on Tas and Gag expression compared to the control group (**Figures 8F, G**). These results of luciferase reporting experiments showed that LTR (3.1-fold change) and IP (3.1-fold change) promoter activities were significantly reduced in the TBC1D16 (424-635) overexpression group (**Figure 8H**, *p* < 0.001). In contrast, when Rab5C was knocked down, no effect of TBC1D16 (424-635) on LTR and IP promoter activation was detected (**Figure 8H**). These results indicated that the interaction between TBC1D16 and Rab5C was essential to inhibit PFV replication, and the TBC domain could inhibit PFV replication independently.

### TBC1D16 Promotes the PFV-Induced Type I Interferon (IFN) Response

It is reported that TBC1D23 acts as a Rab-GAP to regulate innate immunity signaling, strongly inhibiting multiple Toll-like receptor (TLR) and Dectin signaling pathways (52). To determine whether TBC1D16 is involved in regulating innate immunity signaling, we detected the effect of TBC1D16 on PFV-induced IFN signaling. The results showed that overexpression of TBC1D16 enhanced the PFV-triggered activation of IFN-β promoter in HEK293T cells (**Figure S3A**, *p* < 0.01). And when endogenous TBC1D16 was knocked down through shRNA in HEK293T cells, PFV-induced activation of IFN-β promoter was reduced (*p* < 0.001), whereas exogenous expression of TBC1D16 restored the activation of IFN-β promoter (**Figure S3A**, *p* < 0.01). Similarly, the result of ELISA showed that overexpression TBC1D16 also promoted PFV-induced IFN-β production (**Figure S3B**, *p* < 0.01). Moreover, in THP-1 cells which knocked down TBC1D16 with shRNA, the production of IFN-β was significantly reduced (**Figure S3B**, *p* < 0.001), while exogenous expression of TBC1D16 restored the production of IFN-β (**Figure S3B**, *p* < 0.001). These results indicated that TBC1D16 promoted the production of PFV-induced IFN-β.

To investigate whether TBC1D16 also promoted the PFV-induced IFN-I signaling, we determined the transcription of the IFN-β downstream genes in THP-1 cells. As expected, the results of QPCR shown that the transcription of downstream genes such as *IFNB1*, *ISG15*, *CXCL10* and *CCL5* induced by PFV was markedly enhanced in THP-1 cells overexpression TBC1D16 compared with control (pCMV-Flag) (**Figure S3C**, **p < 0.01 or ***p < 0.001). In contrast, when TBC1D16 in THP-1 cells was knocked down with shRNA, the transcription of *IFNB1*, *ISG15*, *CXCL10* and *CCL5* induced by PFV was significantly reduced (**Figure S3D**, *p < 0.05, **p < 0.01 or ***p < 0.001). We also tested the effect of TBC1D16 on PFV-induced IFN downstream genes in HT1080. The results showed that, compared with the control (pCMV-Flag), overexpression of TBC1D16 significantly enhanced PFV-induced transcription of *IFNB1*, *ISG15*, *CXCL10* and *CCL5* in HT1080 cells (**Figure S3E**, *p* < 0.001). In contrast, when TBC1D16 in HT1080 cells was knocked down with shRNA, the transcription of *IFNB1*, *ISG15*, *CXCL10* and *CCL5* induced by PFV was significantly reduced (**Figure S3F**, *p* < 0.001). These results indicated that TBC1D16 is involved in the regulation of PFV-induced IFN-β production and the transcription of downstream genes, and this may be one of the mechanisms by which TBC1D16 inhibits PFV replication.

## Discussion

In this study, we observed that TBC1D16 reduced the transcription and translation of Tas and Gag in PFV and suppress the Tas-dependent transactivation of LTR and IP promoters. Silencing TBC1D16 in HEK293T and HT1080 cells enhanced PFV replication. Moreover, the conserved amino acid residues R494 and Q531 in TBC1D16 are essential for inhibiting PFV replication. The deletion of Rab5C, a member of Rab GTPases, reduced the inhibitory effect of TBC1D16 on PFV replication, indicating that Rab5C was the target of TBC1D16 in reducing PFV replication. And the interaction between TBC1D16 and Rab5C is important for reducing PFV replication. In addition, we also found that TBC1D16 promoted the PFV-induced IFN-β production and the transcription of downstream genes.

TBC proteins function as GAPs for small Rab GTPase, which can promote the hydrolysis of Rab-GTP to Rab-GDP to regulation of specific intracellular trafficking pathways (49). TBC proteins also play an important role in different cellular functions, and its defects are closely associated with numerous disease processes (52, 53). Substantial evidence accumulated in recent years has highlighted that TBC proteins as critical players in antiviral immunity. Overexpression of TBC1D20 reduces the infectivity of HIV-1 virus particle-like particles (VLPs) (25), and TBC1D20 can regulate HCV replication by interacting with the HCV nonstructural protein NS5A (26–28). TBC1D16 belongs to the TBC protein family, and the current understanding of it mainly focuses on its role in different diseases. TBC1D16 is highly expressed in epithelial ovarian cancer (EOC) (54). Changes in methylation modification of the TBC1D16 gene are associated with the progression and metastasis of multiple types of tumors (55). However, research on TBC1D16 in terms of anti-viral activity, especially in affecting PFV replication, has not been reported. In this study, we found that TBC1D16 can inhibit the PFV replication and the expression of Tas and Gag. This suggests that TBC1D16 might be another TBC protein involved in the regulation of viral replication in addition to TBC1D20, and might be the first TBC protein found to be involved in the regulation of PFV replication.

TBC proteins are considered to be GAP of Rab-GTPase and acted as negative regulators of Rab GTPases (56). Moreover, TBC proteins accelerate GTP hydrolysis by a similar dual-finger mechanism including the “arginine finger” from the IxxDxxR motif and a “glutamine finger” from the YxQ motif (57). According to our studies, the TBC domain of TBC1D16 can inhibit PFV replication independently, with R494 and Q531 being critical for inhibition of PFV replication (**Figures 8F–H**). Further studies indicated that R494 and Q531 of TBC1D16 were indispensable for its interaction with Rab5C (**Figure 8A**). Our data suggested that we not only identified TBC1D16 as an inhibitor of PFV, but also further identified the key amino acid sites of TBC1D16 in inhibiting PFV replication.

Rab GTPases are the largest family of small GTPases and have a unique role in cell type-specific or tissue-specific membrane transport events (58). Rab5C belongs to Rab GTPases and is an essential component of the secretory/endocytic pathway (59). The study of how the subcellular transport machinery affects innate immunity signaling is a new field that has recently gained much attention (60). Recently, Rab5C has also been reported to participate in the regulation of the virus life cycle. It was reported that Rab5C could play an important role in the lymphocytic choriomeningitis virus (LCMV) life cycle (61), and the LCMV matrix protein could target Rab5c-positive membranes for preassembly of virus particles prior to budding (62). However, to date, there have been no reports of Rab5C inhibiting viral replication. In this study, we found that Rab5C could reduce the transcription and expression of Tas and Gag during PFV infection, and TBC1D16 acted as the GAP of Rab5C in this process.

The innate immune system plays a critical role in controlling retroviral infections, such as screening of interferon-stimulated genes (ISGs) for antiretroviral activity reveals that TRIM56 increases the antiretroviral potential of IFN-α (63). And members of the TBC family are involved in the regulation of innate immunity and antiviral immunity, such as TBC1D23 (64) and TBC1D20 (27, 28). However, it is unclear whether TBC1D16 is involved in immune regulation. Our data indicated that exogenous expression of TBC1D16 activated the IFN-β promoter and promoted the production of PFV-induced IFN-β. And the deficiency of TBC1D16 significantly inhibited the transcription of PFV-induced downstream genes, such as *IFNB1*, *ISG15*, *CXCL10* and *CCL5*. These data suggested that TBC1D16 can promote the PFV-induced IFN signaling. PFV has a complex genome structure and the longest genome, which can maintain a long-term latent infection in the host and is non-pathogenic. However, the mechanism of latent infection of PFV remains unclear and the effect of TBC1D16 on regulating PFV latency has not been investigated so far. We speculated that TBC1D16 might restrict PFV replication by affecting the IFN response induced by PFV, thereby promoting the latent infection of PFV. TBC1D16 regulated the PFV-induced IFN response might provide new insights for the establishment of PFV latency.

In order to further study the mechanism of TBC1D16 inhibiting PFV replication, we also tested the interaction of TBC1D16 with PFV Tas, Gag and Bet. Unfortunately, we did not find a direct interaction between TBC1D16 or Rab5C and Tas, Gag and Bet (**Figure S4**). However, it cannot be excluded that TBC1D16 or Rab5C exerts its inhibitory function by interacting with other molecules of PFV, such as Env and Pol. We also speculated that these proteins might affect PFV replication through other mechanisms, such as regulating IFN-I upstream or downstream signals instead of directly interacting with PFV structural proteins, and this possibility requires further study.

## Data Availability Statement

The original contributions presented in the study are included in the article/**Supplementary Material**. Further inquiries can be directed to the corresponding author.

## Author Contributions

JYa and WL conceptualized the study. JYa, YZ, and PY worked on the data curation and formal analysis. JYa was responsible for writing, reviewing, and editing the manuscript. JYa, YZ, PY, SW, SH, JYi, BP, ZL, YS, XH, and WL worked on the investigation. JYa, YZ, PY, SW, JYi, and WL worked on the methodology. SH, JYi, BP, XH, and WL administered the project. WL was responsible for the resources. JYa, YZ, and SH were responsible for the software. XH, BP, and WL supervised the study. JYa and WL validated the study. JYa wrote the original draft, and all of the authors commented on previous versions of the manuscript. All authors contributed to the article and approved the submitted version.

## Funding

This work was supported by WL under grants from the National Natural Science Foundation of China (No. 81371790 and No. 52073022), the Fundamental Research Funds for the Shenzhen Science and Technology Innovation Committee (No. JCYJ20170818143952175), the Central Universities of China and the Translational Medical Research Fund of Wuhan University School of Medicine (2042018gf0034, 2042017kf0240), the Creative Research Groups of the Hubei Natural Science Foundation (No. 2017CFA017) and PY under grants from the Hubei Provincial Natural Science Foundation (2020CFB294).

## Conflict of Interest

The authors declare that the research was conducted in the absence of any commercial or financial relationships that could be construed as a potential conflict of interest.

## Supplementary Material

Supplementary Figure 1 TBC1D16 inhibited the transcription of PFV Tas and Gag in a dose dependent way. **(A)** HEK293T cells seeded in 12-well plates were transfected with pCMV-Flag or pCMV-Flag-TBC1D16 (0 ~ 1.5 µg). After 24 h of transfection, the cells were infected with PFV for 48 h and then the mRNA expression changes of Tas and Gag were detected by qPCR. **(B)** HT1080 cells seeded in 12-well plates were transfected with pCMV-Flag or pCMV-Flag-TBC1D16 (0 ~ 1.5 µg). After 24 h of transfection, the cells were infected with PFV for 48 h and then the mRNA expression changes of Tas and Gag were detected by qPCR.

Supplementary Figure 2 R494 and Q531 in the TBC domain are important for the enrichment of TBC1D16 on the PFV LTR and IP promoters. **(A)** pCMV-Flag-TBC1D16-R431A (3 μg) and pGL3-PFV-LTR-luc (3 μg) or pGL3-PFV-IP-luc (3 μg) were cotransfected into HEK293T cells for 48 h, and a ChIP assay was used to detect the enrichment of TBC1D16-R431A on the PFV LTR and IP promoters. ChIP-qPCR data were normalized by the percent input method (%input with IgG as control). The data are presented as the means ± SD (***p* < 0.01 and ****p* < 0.001). **(B)** pCMV-Flag-TBC1D16-R494A (3 μg) and pGL3-PFV-LTR-luc (3 μg) or pGL3-PFV-IP-luc (3 μg) were cotransfected into HEK293T cells for 48 h, and a ChIP assay was used to detect the enrichment of TBC1D16-R494A on the PFV LTR and IP promoters. ChIP-qPCR data were normalized by the percent input method (%input with IgG as control). The data are presented as the means ± SD. **(C)** pCMV-Flag-TBC1D16-R531A (3 μg) and pGL3-PFV-LTR-luc (3 μg) or pGL3-PFV-IP-luc (3 μg) were cotransfected into HEK293T cells for 48 h, and a ChIP assay was used to detect the enrichment of TBC1D16-Q531A on the PFV LTR and IP promoters. ChIP-qPCR data were normalized by the percent input method (%input with IgG as control). The data are presented as the means ± SD. **(D)** pCMV-Flag-TBC1D16-R431AR494AQ531A (3 μg) and pGL3-PFV-LTR-luc (3 μg) or pGL3-PFV-IP-luc (3 μg) were cotransfected into HEK293T cells for 48 h, and a ChIP assay was used to detect the enrichment of TBC1D16-R431AR494AQ531A on the PFV LTR and IP promoters. ChIP-qPCR data were normalized by the percent input method (%input with IgG as control). The data are presented as the means ± SD.

Supplementary Figure 3 TBC1D16 promotes the PFV-induced IFN-β signaling. **(A)** Luciferase assay analyzing *IFNB1* promoter activity in HEK293T cells. HEK293T cells were transfected with a plasmid encoding an IFN-β firefly luciferase reporter (IFN-β–Luc) along with pCMV-Flag-TBC1D16 or shRNA targeting TBC1D16 (400 ng each) for 24h and used pCMV-Flag or shControl as control. In another group, the TBC1D16 specific shRNA and pCMV-Flag-TBC1D16 were contransfected in HEK293T cells for 24h. Luciferase reporter activity is normalized to that of renilla luciferase (***p* < 0.01, ****p* < 0.001). **(B)** Enzyme-linked immunosorbent assay (ELISA) of  IFN-β in THP-1 cells. THP-1 cells were seeded in 96-well plate for 24 h, and then the cells transfected with pCMV-Flag-TBC1D16 or TBC1D16 specific shRNA. In the other group, TBC1D16-specific shRNA and pCMV-Flag-TBC1D16 were co-transfected in THP-1 cells. After 24 h of transfection, all cells were infected with PFV for 48 h (the uninfected cells as control). Measure the concentration of IFN-β in the culture supernatant with ahuman IFN-β ELISA kit. (**p <0.01, ***p <0.001). **(C, D)** Effects of exogenous expression TBC1D16 or TBC1D16 deficiency on PFV-induced transcription of downstream genes in THP-1 cells. THP-1 cells were transfected with pCMV-Flag-TBC1D16 or shRNA targeting TBC1D16 (1.5 μg each) for 24h and used pCMV-Flag or shControl as control. And then all the cells were uninfected or infected with PFV for a certain period of time. qPCR was used to detect the expression of *IFNB, ISG15, CXCL10, CCL5* in THP-1 cells. **(E, F)** Effects of exogenous expression TBC1D16 or TBC1D16 deficiency on PFV-induced transcription of downstream genes in HT1080 cells. HT1080 cells were transfected with pCMV-Flag-TBC1D16 (1.5 μg each) or shRNA targeting TBC1D16 (1.5 μg each) for 24h and used pCMV-Flag or shControl as control. And then all the cells were uninfected or infected with PFV for a certain period of time. qPCR was used to detect the expression of *IFNB, ISG15, CXCL10, CCL5* in HT1080 cells.

Supplementary Figure 4 There is no direct interaction between PFV and TBC1D16 or Rab5C. **(A)** pCMV-Flag-TBC1D16 overexpressing plasmid (3 μg) was cotransfected with pCMV-Myc-Tas (3 μg), pCMV-His-Gag (3 μg) or pCMV-HA-Bet (3 μg) into HEK293T cells for 48 h. Co-immunoprecipitation and immunoblot analysis were used to detect the interaction between TBC1D16 and Tas, Gag and Bet of PFV in HEK293T cells. **(B)** The pCMV-HA-Rab5C overexpressing plasmid (3 μg) was cotransfected with pCMV-Myc-Tas (3 μg), pCMV-His-Gag (3 μg) or pCMV-His-Bet (3 μg) into HEK293T cells for 48 h. Co-immunoprecipitation and immunoblot analysis were used to detect the interaction between Rab5C and Tas, Gag and Bet of PFV in HEK293T cells.
