# Supplementary figures and images for: Novel Host Protein TBC1D16, a GTPase Activating Protein of Rab5C, Inhibits Prototype Foamy Virus Replication
Source: Front Immunol. 2021 Jul 22;12:658660. doi: 10.3389/fimmu.2021.658660 (PMC8339588; doi:10.3389/fimmu.2021.658660)

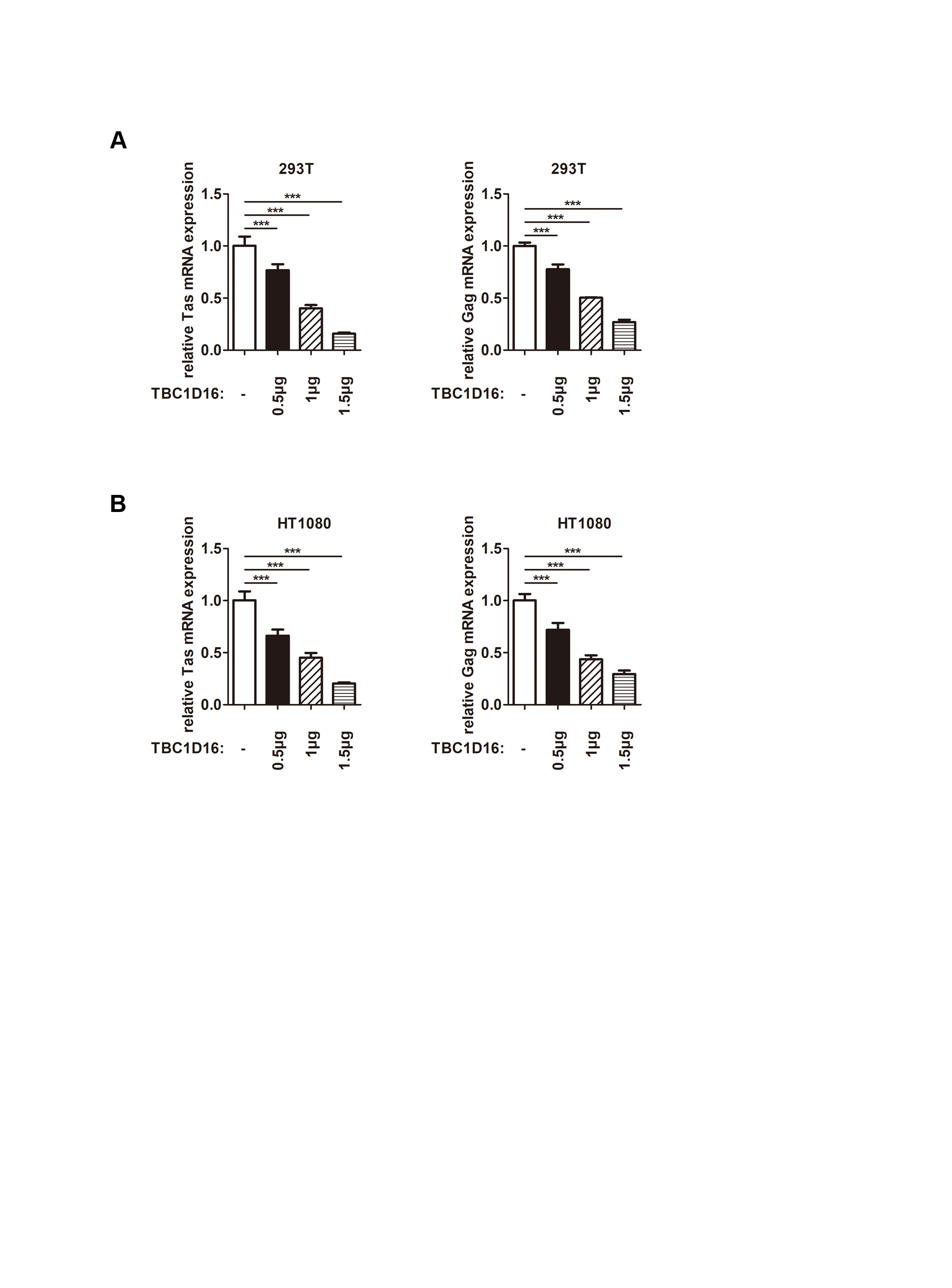

Supplement: Supplementary Figure 1 — TBC1D16 inhibited the transcription of PFV Tas and Gag in a dose dependent way. (A) HEK293T cells seeded in 12-well plates were transfected with pCMV-Flag or pCMV-Flag-TBC1D16 (0 ~ 1.5 µg). After 24 h of transfection, the cells were infected with PFV for 48 h and then the mRNA expression changes of Tas and Gag were detected by qPCR. (B) HT1080 cells seeded in 12-well plates were transfected with pCMV-Flag or pCMV-Flag-TBC1D16 (0 ~ 1.5 µg). After 24 h of transfection, the cells were infected with PFV for 48 h and then the mRNA expression changes of Tas and Gag were detected by qPCR. [file Image_1.tif]

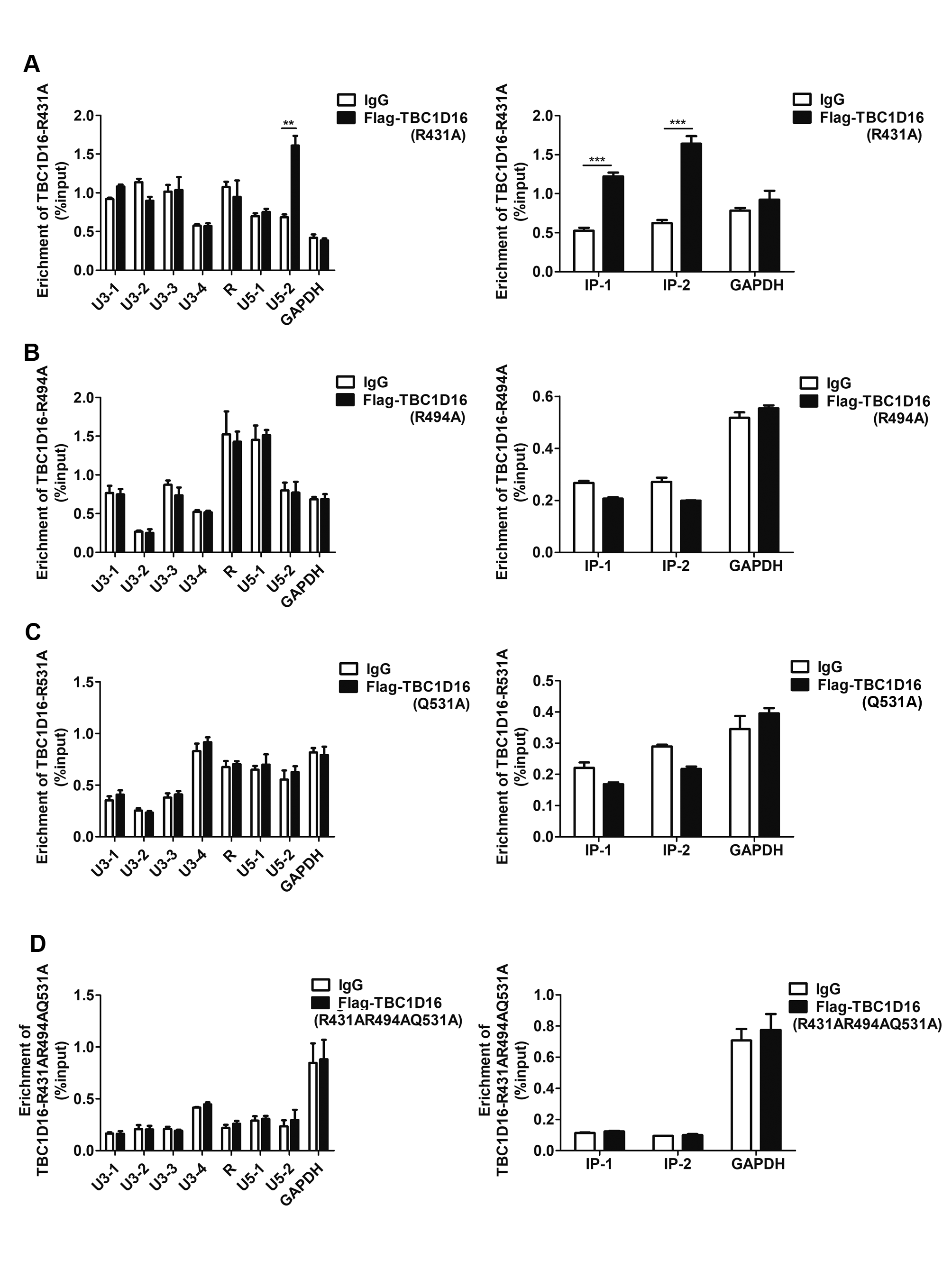

Supplement: Supplementary Figure 2 — R494 and Q531 in the TBC domain are important for the enrichment of TBC1D16 on the PFV LTR and IP promoters. (A) pCMV-Flag-TBC1D16-R431A (3 μg) and pGL3-PFV-LTR-luc (3 μg) or pGL3-PFV-IP-luc (3 μg) were cotransfected into HEK293T cells for 48 h, and a ChIP assay was used to detect the enrichment of TBC1D16-R431A on the PFV LTR and IP promoters. ChIP-qPCR data were normalized by the percent input method (%input with IgG as control). The data are presented as the means ± SD (**p < 0.01 and ***p < 0.001). (B) pCMV-Flag-TBC1D16-R494A (3 μg) and pGL3-PFV-LTR-luc (3 μg) or pGL3-PFV-IP-luc (3 μg) were cotransfected into HEK293T cells for 48 h, and a ChIP assay was used to detect the enrichment of TBC1D16-R494A on the PFV LTR and IP promoters. ChIP-qPCR data were normalized by the percent input method (%input with IgG as control). The data are presented as the means ± SD. (C) pCMV-Flag-TBC1D16-R531A (3 μg) and pGL3-PFV-LTR-luc (3 μg) or pGL3-PFV-IP-luc (3 μg) were cotransfected into HEK293T cells for 48 h, and a ChIP assay was used to detect the enrichment of TBC1D16-Q531A on the PFV LTR and IP promoters. ChIP-qPCR data were normalized by the percent input method (%input with IgG as control). The data are presented as the means ± SD. (D) pCMV-Flag-TBC1D16-R431AR494AQ531A (3 μg) and pGL3-PFV-LTR-luc (3 μg) or pGL3-PFV-IP-luc (3 μg) were cotransfected into HEK293T cells for 48 h, and a ChIP assay was used to detect the enrichment of TBC1D16-R431AR494AQ531A on the PFV LTR and IP promoters. ChIP-qPCR data were normalized by the percent input method (%input with IgG as control). The data are presented as the means ± SD. [file Image_2.tif]

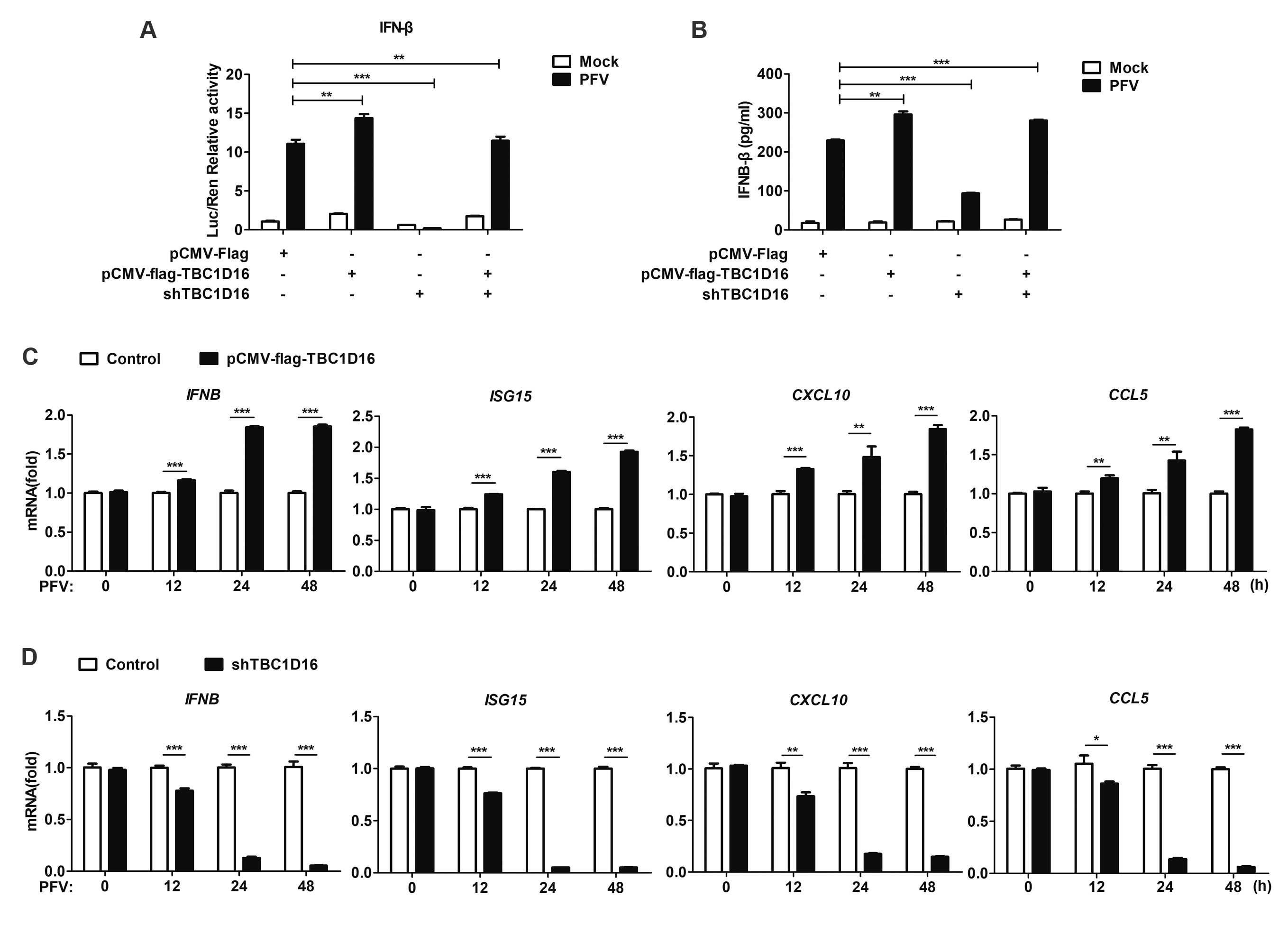

Supplement: Supplementary Figure 3 — TBC1D16 promotes the PFV-induced IFN-β signaling. (A) Luciferase assay analyzing IFNB1 promoter activity in HEK293T cells. HEK293T cells were transfected with a plasmid encoding an IFN-β firefly luciferase reporter (IFN-β–Luc) along with pCMV-Flag-TBC1D16 or shRNA targeting TBC1D16 (400 ng each) for 24h and used pCMV-Flag or shControl as control. In another group, the TBC1D16 specific shRNA and pCMV-Flag-TBC1D16 were contransfected in HEK293T cells for 24h. Luciferase reporter activity is normalized to that of renilla luciferase (**p < 0.01, ***p < 0.001). (B) Enzyme-linked immunosorbent assay (ELISA) of IFN-β in THP-1 cells. THP-1 cells were seeded in 96-well plate for 24 h, and then the cells transfected with pCMV-Flag-TBC1D16 or TBC1D16 specific shRNA. In the other group, TBC1D16-specific shRNA and pCMV-Flag-TBC1D16 were co-transfected in THP-1 cells. After 24 h of transfection, all cells were infected with PFV for 48 h (the uninfected cells as control). Measure the concentration of IFN-β in the culture supernatant with ahuman IFN-β ELISA kit. (**p <0.01, ***p <0.001). (C, D) Effects of exogenous expression TBC1D16 or TBC1D16 deficiency on PFV-induced transcription of downstream genes in THP-1 cells. THP-1 cells were transfected with pCMV-Flag-TBC1D16 or shRNA targeting TBC1D16 (1.5 μg each) for 24h and used pCMV-Flag or shControl as control. And then all the cells were uninfected or infected with PFV for a certain period of time. qPCR was used to detect the expression of IFNB, ISG15, CXCL10, CCL5 in THP-1 cells. (E, F) Effects of exogenous expression TBC1D16 or TBC1D16 deficiency on PFV-induced transcription of downstream genes in HT1080 cells. HT1080 cells were transfected with pCMV-Flag-TBC1D16 (1.5 μg each) or shRNA targeting TBC1D16 (1.5 μg each) for 24h and used pCMV-Flag or shControl as control. And then all the cells were uninfected or infected with PFV for a certain period of time. qPCR was used to detect the expression of IFNB, ISG15, CXCL10, CCL5 in HT [file Image_3.tif]

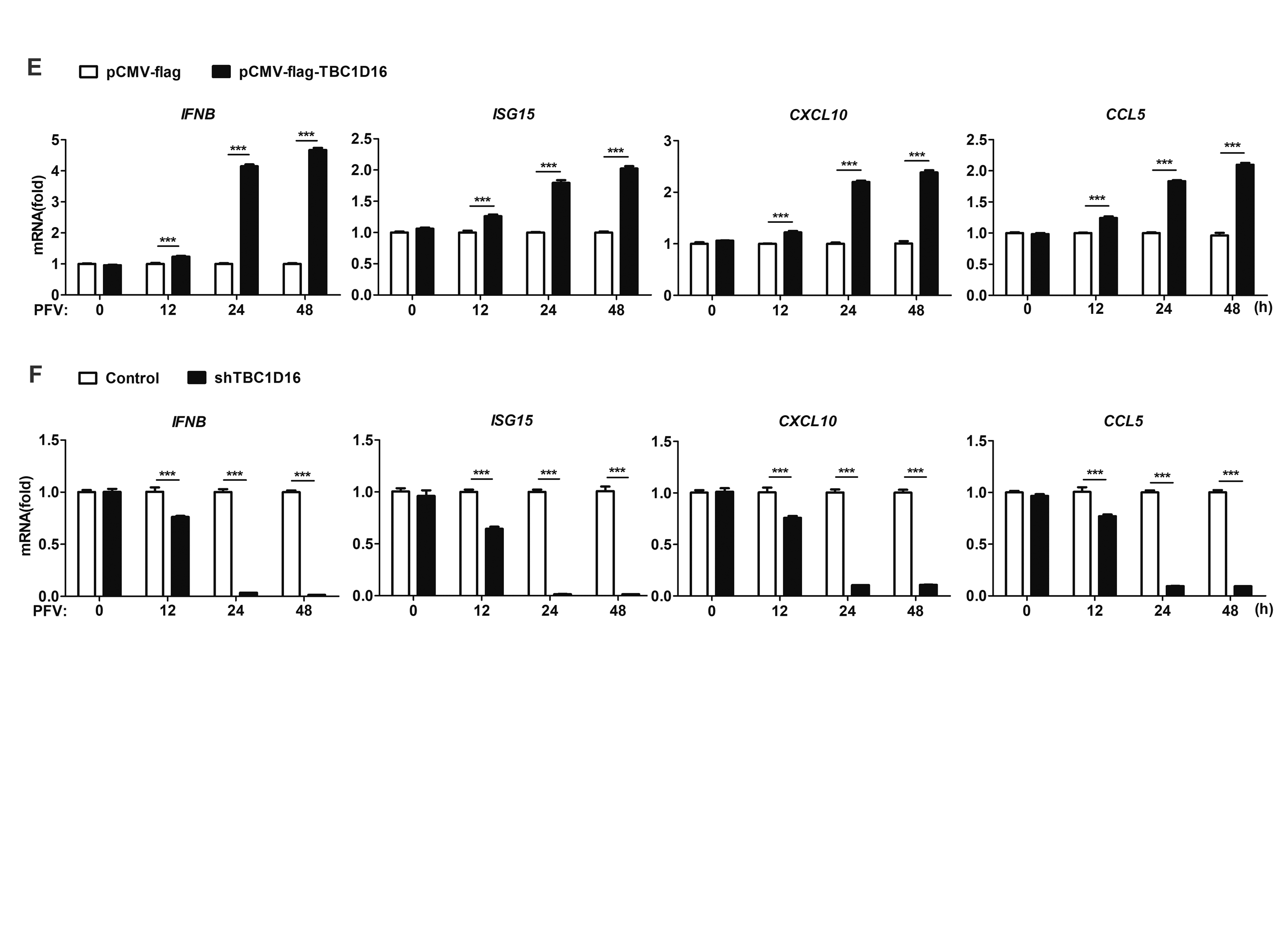

Supplement: Supplementary Figure 4 — There is no direct interaction between PFV and TBC1D16 or Rab5C. (A) pCMV-Flag-TBC1D16 overexpressing plasmid (3 μg) was cotransfected with pCMV-Myc-Tas (3 μg), pCMV-His-Gag (3 μg) or pCMV-HA-Bet (3 μg) into HEK293T cells for 48 h. Co-immunoprecipitation and immunoblot analysis were used to detect the interaction between TBC1D16 and Tas, Gag and Bet of PFV in HEK293T cells. (B) The pCMV-HA-Rab5C overexpressing plasmid (3 μg) was cotransfected with pCMV-Myc-Tas (3 μg), pCMV-His-Gag (3 μg) or pCMV-His-Bet (3 μg) into HEK293T cells for 48 h. Co-immunoprecipitation and immunoblot analysis were used to detect the interaction between Rab5C and Tas, Gag and Bet of PFV in HEK293T cells. [file Image_4.tif]

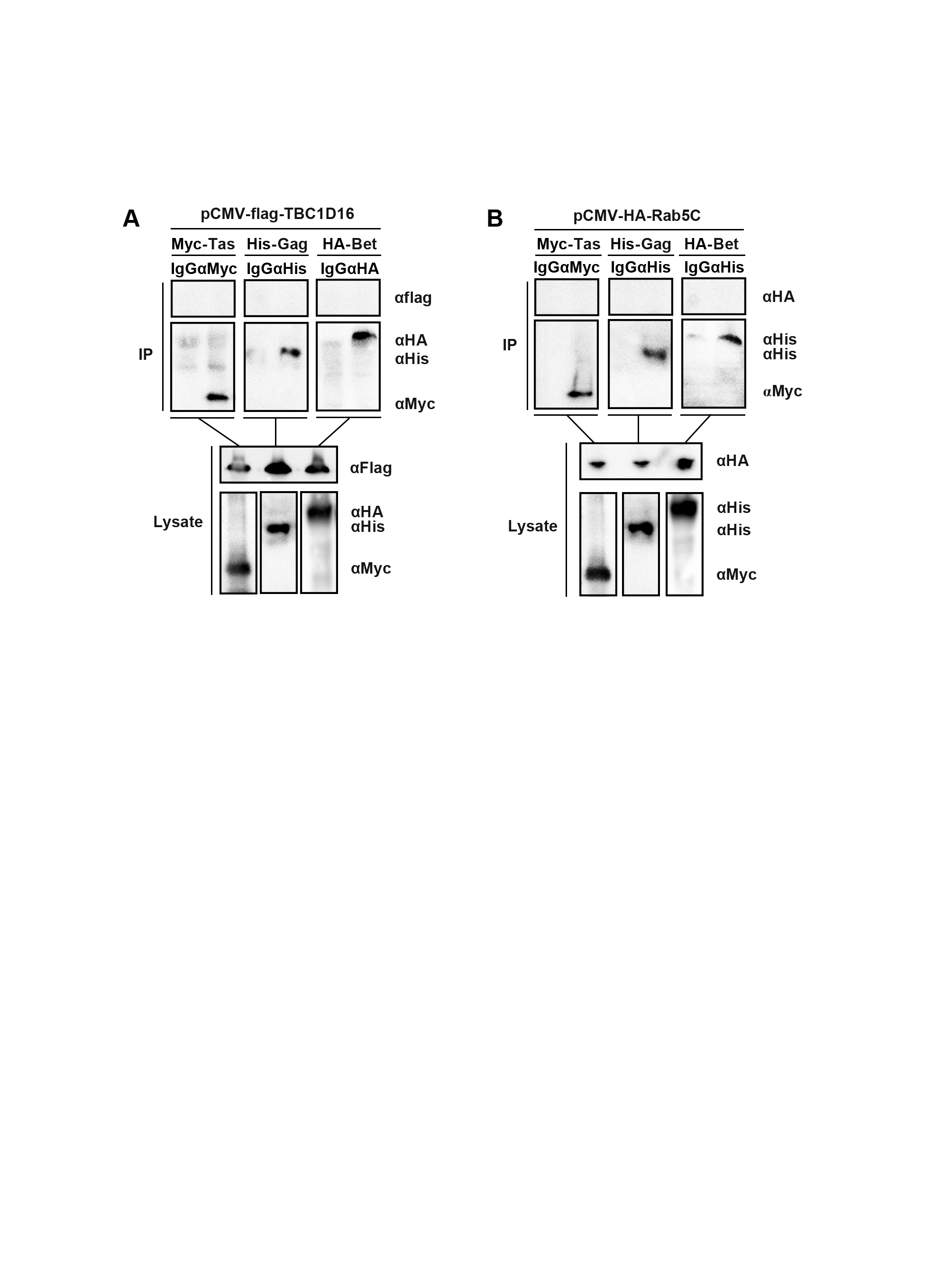

Supplement: Supplementary file 5 [file Image_5.tif]
