# Supplementary material for: Novel Host Protein TBC1D16, a GTPase Activating Protein of Rab5C, Inhibits Prototype Foamy Virus Replication
Source: Front Immunol. 2021 Jul 22;12:658660. doi: 10.3389/fimmu.2021.658660 (PMC8339588; doi:10.3389/fimmu.2021.658660)
Supplement: Supplementary file 6 [file Table_1.docx]

**Table S1 Primers for PCR amplification**

| **Description** | **Primers** |
| --- | --- |
| TBC1D16-F | 5’-GGAATTCATGCGGAGGATGGAAACACT-3’ |
| TBC1D16-R | 5’-GAAGATCTCTATCTGCGGAAGCCGAAGC-3’ |
| Rab5C-F | 5’-ACGCGTCGACGATGGAACTGAGTTGGAGGTCCCCCT-3’ |
| Rab5C-R | 5’-GGGGTACCTCAGTTGCTGCAGCACTGGCTCC-3’ |
| Rab4A-F | 5’-GGAATTCATATGTCGCAGACGGCCATGTCC-3’ |
| Rab4A-R | 5’-GGGGTACCCTAACAACCACACTCCTGAGCGTTC-3’ |
| TBC1D16 (1-212)-F | 5’-CCCAAGCTTATGTCTCTGGGCCGCCTC-3’ |
| TBC1D16 (1-212)-R | 5’-GGGGTACCCTAGCCATCCTCCTCCCCGGC-3’ |
| TBC1D16 (212-265)-F | 5’-CCCAAGCTTTCTTTGGAACTGTCAGCCGAGGG-3’ |
| TBC1D16 (212-265)-R | 5’-GGGGTACCCTAGGAGCTGGACGGGGGGCT-3’ |
| TBC1D16 (424-635)-F | 5’-CCCAAGCTTGGTATTGATGTGTCAATCC-3’ |
| TBC1D16 (424-635)-R | 5’-GGGGTACCCTAGTAGTGGGCCCAGCAGGCCTC-3’ |
| TBC1D16 (635-767)-F | 5’-CCCAAGCTTCAGACGGACTACTTCCACCTTTTCA-3’ |
| TBC1D16 (635-767)-R | 5’-GGGGTACCCTATCTGCGGAAGCCGAAGCC-3’ |
| TBC1D16 (R431A)-F | 5’-GCGGTATTGATGTGTCAATC**GCA**GGGGAGGTCT |
|  | GGCCCTTCCT-3’ |
| TBC1D16 (R431A)-R | 5’-AGGAAGGGCCAGACCTCCCC**TGC**GATTGACACA |
|  | TCAATACCGC-3’ |
| TBC1D16 (R494A)-F | 5’-CTGTGGACAAAGACGTGGTC**GCA**ACAGATCGGA |
|  | ACAACCAGTT-3’ |
| TBC1D16 (R494A)-R | 5’-AACTGGTTGTTCCGATCTGT**TGC**GACCACGTCTT |
|  | TGTCCACAG-3’ |
| TBC1D16 (Q531A)-F | 5’-CTGCCGTCGGCTATTCC**GCA**GGGATGTCGGACC |
|  | TGGT-3’ |
| TBC1D16 (Q531A)-R | 5’-ACCAGGTCCGACATCCC**TGC**GGAATAGCCGACG |
|  | GCAG-3’ |
|  |  |

**Note:** Bold indicates that the conserved amino acid residues (R431, R494 and Q531) of TBC1D16 mutated into alanine.
