# Supplementary material for: Novel Host Protein TBC1D16, a GTPase Activating Protein of Rab5C, Inhibits Prototype Foamy Virus Replication
Source: Front Immunol. 2021 Jul 22;12:658660. doi: 10.3389/fimmu.2021.658660 (PMC8339588; doi:10.3389/fimmu.2021.658660)
Supplement: Supplementary file 7 [file Table_2.docx]

**Table S2.** **Primer sequences for qPCR and ChIP**

| **Description** | **Primers** |
| --- | --- |
| qPCR-*actin*-F | 5’-CACGATGGAGGGGCCGGACTCATC-3’ |
| qPCR-*actin*-R | 5’-TAAAGACCTCTATGCCAACACAGT-3’ |
| qPCR-*gag*-F | 5'-AATAGCGGGCGGGGACGACA-3' |
| qPCR-*gag*-R | 5'-ATTGCCACGCACCCCAGAGC-3' |
| qPCR-*tas*-F | 5'-GGAACAATCAGATACTGACCCT-3' |
| qPCR-*tas*-R | 5'-CCAACTTCAGGATCCCATCTT-3' |
| ChIP-GAPDH-pro-F | 5’-GAAGGTGAAGGTCGGAGTCA-3’ |
| ChIP-GAPDH-pro-R | 5’-CCCATACGACTGCAAAGACC-3’ |
| ChIP-LTR-U3-1-F (76-92) | 5’-ACCCAAATACTCCTGC-3’ |
| ChIP-LTR-U3-1-R (134-151) | 5’-TAGCGAATGAGGACAATT-3’ |
| ChIP-LTR-U3-2-F (151-166) | 5’-ACTCCCTCTGACATCC-3’ |
| ChIP-LTR-U3-2-R (353-368) | 5’-TTTTCGGTGTCTGTCA-3’ |
| ChIP-LTR-U3-3-F (355-370) | 5’-AAGCCACAGACAGTAA-3’ |
| ChIP-LTR-U3-3-R (574-589) | 5’-TGCATCCCACTGTTCT-3’ |
| ChIP-LTR-U3-4-F (573-588) | 5’-CACGTAGGGTGACAAG-3’ |
| ChIP-LTR-U3-4-R (784-800) | 5’-GAGAAGTGATGAGCGAC-3’ |
| ChIP-LTR-R-F (767-782) | 5’-TAGATTGTACGGGAGC-3’ |
| ChIP-LTR-R-R (951-966) | 5’- TGGCTGAACTAAGCTC-3’ |
| ChIP-LTR-U5-1-F (947-963) | 5’-ATAAACCGACTTGATTC-3’ |
| ChIP-LTR-U5-1-R (1036-1051) | 5’-CACTAGATGTCTCCCT-3’ |
| ChIP-LTR-U5-2-F (1072-1090) | 5’-CTTAAATGATGTAACTCCT-3’ |
| ChIP-LTR-U5-2-R (1050-1167) | 5’-TACAAATAAACCCGACTT-3’ |
| ChIP-IP-1-F (9019-9038) | 5’-CTGGACTTTAAAAGGCCACT-3’ |
| ChIP-IP-1-R (9091-9108) | 5’-AACCAAATGTGGTAATCT-3’ |
| ChIP-IP-2-F (9103-9120) | 5’-TTTGGTTGGAATTATTGC-3’ |
| ChIP-IP-2-F (9179-9196) | 5’-AGCTTTTGCTCTTTCAAT-3’ |
| qPCR-*TBC1D16*-F | 5’-AGATGCAGCTCAAAGACCAGG-3’ |
| qPCR-*TBC1D16*-R | 5’-GAAAATGGCCTTCCGCAGC-3’ |
| qPCR-*Rab5C*-F | 5’-GAGCCTCTGAAGCTGTCACT-3’ |
| qPCR-*Rab5C*-R | 5’-GGACCTCCAACTCAGTTCCAT-3’ |
| qPCR-*Rab4A*-F | 5’-ATGCAGGAACTGGCAAATCTT-3’ |
| qPCR-*Rab4A*-R | 5’-TCACGGACCTGAATCGTTCTT-3’ |
| qPCR-*IFNB*-F | 5’-TTGTTGAGAACCTCCTGGCT-3’ |
| qPCR-*IFNB*-R | 5’-TGACTATGGTCCAGGCACAG-3’ |
| qPCR-*ISG15*-F | 5’-GGTGGACAAATGCGACGAAC-3’ |
| qPCR-*ISG15*-R | 5’-TCGAAGGTCAGCCAGAACAG-3’ |
| qPCR-*CXCL10*-F | 5’-CCACGTGTTGAGATCATTGCTAC-3’ |
| qPCR-*CXCL10*-R | 5’-CTGCATCGATTTTGCTCCCC-3’ |
| qPCR-*CCL5*-F | 5’-ATCTGCCTCCCCATATTCCTC-3’ |
| qPCR-*CCL5*-R | 5’-GTCCCTCTCTCTTTGGCATCC-3’ |
| qPCR-*RPS9*-F | 5’-CTTCCGAGTTTCCATGAGCG-3’ |
| qPCR-*RPS9*-R | 5’-ATACTCGCCGATCAGCTTCAG-3’ |
| qPCR-*LYAR*-F | 5’-CTGGGTGGTTCAGAAGCGAA-3’ |
| qPCR-*LYAR*-R | 5’-ACAGCAAACATTGCCTCTCAG-3’ |
| qPCR-*Vps72*-F | 5’-TCTCGGTAGGCGGTATGAGT-3’ |
| qPCR-*Vps72*-R | 5’-CGGATTCCTCTGTAGAACCCC-3’ |
| qPCR-*APLP2*-F | 5’-TCAAGTGTCTCGTGGGTGAA-3’ |
| qPCR-*APLP2*-R | 5’-AATCTTTGTCTCTTTGACTACCGT-3’ |
| qPCR-*ACTR1A*-F | 5’-TCCTCTGCCATGGAGTCCTA-3’ |
| qPCR-*ACTR1A*-R | 5’-GGCCCACATAGTTTGGAAAGC-3’ |
| qPCR-*REPIN1*-F | 5’-GTCTCCAGGGGAGCTCAGT-3’ |
| qPCR-*REPIN1*-R | 5’-CCACTGCTCCCTACAGTGC-3’ |
| qPCR-*APC*-F | 5’-AAGAGAGAGGAGACAAAACCGC-3’ |
| qPCR-*APC*-R | 5’-AGTTCTCCATCTTCAGTGCCTC-3’ |
| qPCR-*PRICKLE1*-F | 5’-ACTTTCTGGGCTCTGGATGGTTC-3’ |
| qPCR-*PRICKLE1*-R | 5’-ACATCAAACAATGGCTGCTCGC-3’ |
| qPCR-*FLNB*-F | 5’-GAAGGATGTTGTGGACCCCAG-3’ |
| qPCR-*FLNB*-R | 5’-ACTGGGAATCCGTGTCGTC-3’ |
|  |  |
